# Development and validation of a domain-specific scale of founder characteristics associated with startup success

**DOI:** 10.1371/journal.pone.0351970

**Published:** 2026-06-26

**Authors:** Irena C. Danilovska, Manousos Klados, Catherine Preston, Nick E. Barraclough

**Affiliations:** 1 Department of Psychology, University of York, Heslington, York, United Kingdom; 2 Department of Psychology, University of York Europe Campus CITY College, Thessaloniki, Greece; Universidade Europeia, Lisboa, PORTUGAL

## Abstract

Early differentiation of startup founders with high success potential remains a central challenge for entrepreneurship research, innovation policy, and capital allocation in high-uncertainty environments. Existing approaches rely largely on broad personality models or intention-based instruments developed primarily to explain entry into entrepreneurship rather than differentiation of founders associated with realized venture success. In this study, we introduce a domain-specific model of founder success characteristics and develop a 31-item Startup Founder Success Scale (SFSS) to differentiate realized success from entrepreneurial intent. The sample (N = 10,007) included startup founders recruited from predefined pools meeting objective funding, revenue, or acquisition benchmarks, as well as corporate managers and aspiring entrepreneurs. Exploratory and confirmatory factor analyses supported six distinct dimensions—Relentless Resilience, Value-Creating Opportunism, Intrinsic Curiosity, Courageous Decision-Making, Strategic Innovativeness, and Transformational Leadership—which together accounted for 71% of the common variance in founder-specific latent trait measures within the scale, indicating strong internal coherence. Group comparisons showed that these dimensions reliably distinguished Successful Startup Founders from Corporate Managers and Aspiring Entrepreneurs, with large effect-size separations (Cohen’s d = 0.83–1.77) exceeding the small-to-moderate effects (d ≈ 0.2–0.5) typically reported for broad personality traits in entrepreneurship research. Conceptually, these findings are consistent with a multi-level framework in which founder-specific characteristics are closely aligned with decision-making under uncertainty and resource orchestration during early venture execution, helping explain why intent-based and general personality models, while valuable for understanding entrepreneurial entry, may be less closely aligned with realized startup success. Practically, the SFSS provides a validated psychometric tool that may support more structured evaluation of founder-related characteristics, targeted training, and resource allocation across venture capital, angel investing, public investment bodies, accelerators, and entrepreneurial education programs. Prospective longitudinal research is required to establish predictive validity for real-world outcomes.

## 1. Introduction

Early differentiation of startup founders more likely to succeed has significant economic and policy implications in entrepreneurial finance. Directing funding, mentorship, and specialized training toward individuals exhibiting founder characteristics associated with realized success may improve the efficiency of resource allocation, entrepreneurial support practices, venture outcomes, investment performance, and exit potential. Yet, existing entrepreneurial personality and intention instruments have rarely been developed or systematically validated using samples of real-world startup founders with objectively verified success outcomes. Most prior models, often theory-driven, underpowered, or based on student samples used as proxies, rely on general entrepreneurial intent or personality frameworks that may not fully capture the domain-specific dispositions associated with realized startup success. This distinction is increasingly recognized within the broader entrepreneurship literature, which has emphasized important differences between innovation-driven entrepreneurship and other forms of entrepreneurial activity in terms of uncertainty, growth dynamics, organizational demands, and economic impact [1]. Consequently, our central research question is whether founder-specific success characteristics provide greater explanatory specificity for distinguishing profiles of startup founders with realized success relative to (a) broad personality models and (b) current entrepreneurial intention and orientation instruments?

For clarity and consistency, we adopt the following operational definitions. “Success” refers to externally verifiable venture outcomes (e.g., funding thresholds, revenue milestones, or acquisition events), rather than subjective or reputational notions of entrepreneurial achievement. A “startup founder” is defined as an individual who initiates and leads a venture designed for scalable, innovation-driven growth, while retaining meaningful strategic decision authority and equity during formative stages. “Founder characteristics” are conceptualized as domain-specific, relatively stable dispositional, cognitive, and behavioral tendencies associated with how founders evaluate asymmetric opportunities, make decisions under uncertainty, orchestrate resources, and mobilize others. The terms “decision-making under uncertainty” and “resource orchestration” refer pragmatically to refer to how founders evaluate ambiguous information, tolerate risk and loss, and mobilize resources over time to support venture development. Finally, we distinguish “broad personality measures” (e.g., Big Five) and “entrepreneurial intention/ orientation instruments” (e.g., IEO, EPS) from “founder-specific models of success characteristics”, namely the Startup Founder Success Scale (SFSS), which targets characteristics associated with realized startup success.

Over the past three years, $1.5 trillion of venture funding has been invested [2], while the global startup economy generated $3 trillion in value from 2017 to mid-2019 and has grown >10% annually, outpacing the broader economy [3]. Startups also drive job creation, unlike many established companies that are reducing employment during economic fluctuations [4,5]. However, startup success remains elusive ─ failure rates exceed 75% [6] and can reach 90% [7]. High-level success ($50 million+ exit) is achieved by only 1.5% of the funded startups [8]. Resources invested in unsuccessful startups do not reliably yield learning benefits [9] and in some cases can contribute to harm for customers, investors, and society at large (cf. cases such as Sam Bankman-Fried, Adam Neumann, Elizabeth Holmes; see also [10,11]).

Suboptimal investment allocation often stems from subjective, network-driven selection that privileges factors like pedigree, geography, or access over merit. When formal assessments are used, they typically draw on broad personality models that do not fully capture founder-specific characteristics associated with startup success. Together, these practice- and model-level limitations may contribute to founder profiles associated with strong venture outcomes being overlooked and resources being allocated inefficiently. Ongoing macroeconomic and geopolitical volatility, coupled with tighter investment funds availability [2], underscores the need for rigorous selection and targeted support of startup founders adept at navigating uncertainty. This objective becomes even more complex given that entrepreneurship is a multifaceted journey, thus, understanding founder characteristics is likely to be critical, as they play a significant role in shaping the culture, direction, and the success of the startup company [12–14].

Recent research in entrepreneurial finance has highlighted the role of personality in investment decisions and funding success [15]. However, many of these studies rely on broad personality or trait models that, while informative, tend to explain a limited proportion of variance [16,17] and exhibit small effect sizes [18]. Prior work has characterized the personality traits of successful entrepreneurs either by assessing them against broad models of personality or by isolating one or few traits. However, these models have primarily been built on previous theories (e.g., [19–21]), where some are underpowered, or validated with convenient samples (e.g., university students [22,23]), often making it difficult to distinguish traits associated with entrepreneurial intent from those more closely linked to founders’ realized success.

For example, the Big Five model, encompassing five broad personality dimensions [24], while predictive of general life outcomes, may not adequately capture success-relevant variation among early-stage startup founders [22,23]. Empirical evidence suggests that Big Five personality traits explain only a limited proportion of variance in entrepreneurial performance outcomes (~10–15% [16,17]), suggesting the need to supplement these models with domain-specific approaches tailored to startup execution. These conventional models capture general dispositional tendencies, which may not align closely with the context-dependent demands of startup entrepreneurship.

Approaches exploring the role of alternative traits for entrepreneurial success like risk propensity (e.g., [25,26]), entrepreneurial self-efficacy (e.g., [27,28]), innovativeness (e.g., [29,30]), proactiveness (e.g., [31]), need for achievement (e.g., [32,33]), resilience (e.g., [34,35]), and locus of control (e.g., [36]) aimed to offer better insight into entrepreneurial achievement. While informative, isolated traits approaches may capture only subsets of the multidimensional demands associated with startup entrepreneurship.

Howard [20] addressed this by introducing the Entrepreneurial Personality Scale (EPS), based on a systematic review [37], aggregating frequently cited traits such as innovativeness, risk-taking propensity, achievement orientation, proactiveness, locus of control, self-efficacy, and autonomy orientation. However, the study relied primarily on ‘general participants’ (n = 1385) and ‘business owners’ (n = 492; 61% female) recruited through online platforms (MTurk and Prolific) rather than objectively verified successful startup founders, and did not report total variance explained, limiting direct comparison at the level of construct coverage. Other entrepreneurial instruments face distinct methodological constraints. Bolton and Lane’s Individual Entrepreneurial Orientation scale (IEO [19]), adapted from the firm-level Lumpkin and Dess’s Entrepreneurial Orientation (EO) framework [13], raises potential level-of-analysis concerns when applied to individuals. While EO explains approximately 24% of variance in organizational contexts [38], the IEO reported to account for 60% of variance using only 10 items. In comparison, Santos et al.’s Entrepreneurial Potential Assessment Inventory (EPAI [21]) focuses primarily on entrepreneurial readiness, motivations, competencies, and social factors, rather than founder-specific success characteristics, while Staniewski’s Successful Entrepreneurship Scale [39] emphasizes knowledge- and skill-based predictors over dispositional traits. Additionally, Bolton and Lane’s and Santos et al.’s scales relied on university student samples, while Staniewski used a relatively small Poland-based sample (n = 294), limiting generalizability. Collectively, these instruments provide useful foundations for understanding entrepreneurial orientation and personality but were not specifically optimized to distinguish founders associated with realized high-growth startup outcomes.

Furthermore, prior entrepreneurship research has often conflated heterogeneous groups under the broad label of ‘entrepreneur’ [22], including students enrolled in entrepreneurship courses (e.g., [19,21], self-employed micro-business owners (e.g., [40]), local farmers (e.g., [41]), tourism workers (e.g., [42]), founders of technology-based ventures (e.g., [43,44]), and business managers (e.g., [45]). Extrapolating findings across these distinct subgroups complicates interpretation and generalization to innovation-driven startup founders. Consequently, most of the literature has not provided a clear operational distinction regarding what constitutes a successful startup founder [18,46].

Crucially, highly successful startup founders must be distinguished from other groups of individuals in parallel professions, like corporate management, or engaged in small-scale entrepreneurship, as distinctive sub-types of businesspeople are likely to exhibit significant behavioural diversity [22,47–50]).

Nevertheless, existing research has predominantly focused on examining the connection between personality traits that are driving the individuals’ inclination to start entrepreneurial ventures, rather than characteristics associated with realized venture outcomes [23]. Traits exhibited by successful startup founders may differ from those associated with entrepreneurial entry [17,51]. In addition, success is inconsistently defined, often with low thresholds for profitability or growth (e.g., [52]) or incomparable metrics [53], and sometimes proxied by long-term firm survival (e.g., [54]), which misaligns with startup aims of rapid scaling and strategic exits (e.g., Merger and Acquisition (M&A) or Initial Public Offering (IPO)). Overall, differences in design choices, samples, and definitions across prior studies complicate cumulative inference and generalizability [22,23].

### 1.1. Conceptual contribution: Bridging entrepreneurial intent and realized success

Prior entrepreneurship research has predominantly focused on *who intends to become an entrepreneur*, rather than *who succeeds as a startup founder*. As a result, much of the literature relies on broad personality frameworks or intent-based instruments that predict entry into entrepreneurship but show limited explanatory power for realized venture success. This creates a persistent theoretical gap between models of entrepreneurial intention and empirical observations of high-variance startup outcomes.

The present study contributes to theory by explicitly addressing this gap. Rather than treating entrepreneurial success as a downstream extension of intent or general personality, we conceptualize a founder-specific model of success characteristics as domain-bound dispositions that shape decision-making under uncertainty and the orchestration of resources in early venture contexts. The SFSS reframes entrepreneurship not as a binary choice to enter, but as a sustained execution problem, characterized by asymmetric risk, incomplete information, and path-dependent decisions over time. By empirically demonstrating that these founder-specific characteristics distinguish successful founders from both aspiring entrepreneurs and corporate managers, the present work extends intent-focused accounts toward a success-relevant and empirically discriminative framework, clarifying why broad personality models may show comparatively lower explanatory specificity in this domain.

In this study, we address prior limitations by advancing a founder-specific model of success characteristics, and by situating this model within a conceptual framework linking founder characteristics to decision-making under uncertainty and resource orchestration. Empirically, we develop the 31-item Startup Founder Success Scale (SFSS), grounded in insights from autobiographies of founders who built highly successful globally scaled companies, mentors, and high-return investors, and validate it with N = 10,007 participants (Successful Startup Founders, Corporate Managers, and Aspiring Entrepreneurs). Successful Startup Founders were recruited from predefined, objectively verifiable founder pools (public funding records, accelerator and investor benchmarks). An Exploratory Factor Analysis (EFA) on a random half-sample yielded a six-dimension structure: Relentless Resilience (30.92%), Value-Creating Opportunism (10.10%), Intrinsic Curiosity (9.35%), Courageous Decision-Making (8.09%), Strategic Innovativeness (6.93%), and Transformational Leadership (5.55%), that jointly explain 71% of the common variance among the measured founder-specific traits. This variance reflects explanatory power within the latent trait structure of the SFSS, indicating strong construct coherence, rather than variance explained in external venture outcomes, which will require prospective longitudinal validation. A second EFA conducted solely on the group of proven successful startup founders supported this structure. The model’s cross-group stability and robustness were subsequently examined using Confirmatory Factor Analysis (CFA) on the remaining 50% of the total sample, indicating interpretable and acceptable model fit across participant groups, alongside large effect-size separations (Cohen’s *d* = 0.83–1.77 [55]) between successful founders and other comparison groups.

Our findings reinforce that entrepreneurial intent does not equate to entrepreneurial success and suggest that only a small subset of aspiring entrepreneurs exhibit the traits associated with realized success. By detailing the development, structure, and group-level comparisons of the SFSS, this study aims to contribute a domain-specific psychometric framework for examining founder characteristics associated with realized startup success. In practical contexts, the SFSS may help inform early-stage VC founder evaluation and entrepreneurial resource-allocation processes by differentiating success-linked founder profiles from entrepreneurial intent alone, while also providing a foundation for future longitudinal studies.

## 2. Materials and methods

### 2.1. Participant selection and recruitment

Scale development analyses were conducted using questionnaire responses from 10,007 participants (n = 10,007; 6,390 males, 3,524 females, 93 others/prefer not to say; mean age = 35.8, SD = 8.8, range 18–59) to items in a questionnaire. Participants were recruited from over 80 countries, with primary representation from Europe and the United States and additional representation from Asia and the Middle East; this geographic diversity supports analysis within a heterogeneous international sample without implying cross-cultural equivalence.

Startup founders were identified via Crunchbase if their companies had raised over USD 1 million across more than one external funding round. In addition, founders were recruited through startup accelerators and angel investor groups if they met predefined financial benchmarks, including either sustained profitability with annual revenues exceeding USD 1 million or completed acquisition transactions exceeding USD 3 million. Freelancers, lifestyle businesses without scalable intent, and owners of purely local, non-scaling firms were excluded. Corporate Managers were recruited via LinkedIn from C-level executives of established firms, including Fortune 500 and FT Europe 500 companies. Aspiring Entrepreneurs were recruited through organizers of well-known startup competitions and incubators and consisted of individuals actively exploring or preparing to enter entrepreneurship but without verified success outcomes. Data were collected between 2015 and 2017 through staged recruitment across multiple founder, executive, and entrepreneurial networks. Recruitment for each participant group occurred in discrete waves, each spanning approximately 6–9 months, reflecting the scale and practical constraints of assembling a large international sample. This approach reduced the likelihood of short-term contextual or event-driven response bias.

Self-identification was used to confirm eligibility within the externally defined criteria. Participants were classified into three groups: Successful Startup Founders (SSF, n = 6,142), Corporate Managers (CM, n = 2,004), and Aspiring Entrepreneurs (AE, n = 1,861). Including all three groups, SSF, CM, and AE, allowed us to capture a broad continuum of entrepreneurial experience and intent, from proven founders to those still at the ideation stage. This design helps reduce the likelihood of overfitting to a single group while enabling comparative analysis of psychological characteristics associated with entrepreneurial success. Comparable group-based designs have been used in previous research on entrepreneurial traits (e.g., [17,19,21,25,33,56,57]), though this is the first study to analyze all three groups within one integrated framework.

We excluded founders whose ventures had not met predefined success benchmarks at the time of data collection, because such outcomes remain indeterminate and may later reverse. Founders in a current state of failure therefore do not constitute a stable comparison group at the opposite end of the success spectrum, as their outcomes may evolve and would introduce ambiguity into success-linked characteristics. Instead, Corporate Managers and Aspiring Entrepreneurs were included as reference groups, representing distinct and comparatively stable levels of entrepreneurial exposure and intent. This design prioritizes construct clarity over representativeness and is intended to identify founder characteristics associated with realized startup success relative to adjacent professional and aspirational groups, rather than to model the full distribution of entrepreneurial trajectories.

Demographic characteristics of the participant sample across groups are summarized in Table 1.

**Table 1:** Demographic characteristics of the participant sample by group.

Group	Successful startup founders (SSF)		Corporate managers (CM)		Aspiring entrepreneurs (AE)		Full sample	
**Dem. characteristic**	**N.**	**%**	**N.**	**%**	**N.**	**%**	**N.**	**%**
**Gender**								
**Male**	4259	69.3	1226	61.2	905	48.6	6390	64.0
**Female**	1855	30.2	750	37.4	918	49.3	3524	35.2
**Not disclosed**	28	0.5	27	1.4	38	2.0	93	0.9
**Age**								
**18-29**	337	5.5	311	15.5	1019	54.7	1667	16.7
**30-39**	1996	32.5	824	41.1	264	14.2	3083	30.8
**40-49**	2853	46.4	705	35.2	440	23.6	3998	39.9
**50-59**	956	15.6	165	8.2	138	7.4	1259	12.6
**Education**								
**High school**	436	7.1	110	5.5	226	12.2	772	7.7
**Bachelor/College**	2755	44.8	851	42.5	1119	60.1	4725	47.2
**Master studies**	2403	39.1	933	46.6	428	23.0	3764	37.6
**Ph.D.**	548	8.9	110	5.5	88	4.7	746	7.4
**Residence**								
**Europe**	2375	38.7	978	48.8	810	43.5	4224	42.2
**USA**	3261	53.1	819	40.8	870	46.7	4889	48.9
**Asia/Middle East**	506	8.2	207	10.3	181	9.7	894	9.0

#### 2.1.1. Ethical considerations and study design.

The study comprised two components with distinct ethical and governance determinations, conducted in accordance with the principles of the Declaration of Helsinki. No minors were involved in either component of the study.

**Retrospective component.** The primary dataset analyzed in this manuscript consisted of questionnaire responses collected prior to the present analyses. On 29 November 2022, the University of York Information Governance Team (Legal Services) determined that formal ethical approval was not required for the analysis and publication of these non-interventional data, which were fully anonymized prior to researcher access. Participants had provided informed consent electronically at the time of original data collection, permitting the use of their fully anonymized responses for research purposes. The data were accessed for research purposes between 5 December 2022 and 15 December 2023. At no point during or after data access did the authors have access to information that could identify individual participants.

**Prospective component.** A separate face- and content-validation study constituted prospective human participant research. Ethical approval for this component was granted by the Departmental Ethics Committee, Department of Psychology, University of York, on 21 April 2023 (reference number 2244). Recruitment took place between 9 May 2023 and 31 May 2023. All participants provided informed consent electronically prior to participation.

### 2.2. Dimensions identification and initial item generation

The present analyses focus on scale development, internal structure, group discrimination, and measurement invariance across participant groups. Predictive validity and cross-cultural calibration are deferred to subsequent validation studies. We adopted an exploratory methodology for identifying questionnaire items as described by Boateng et al. [58], diverging from conventional theory-based approaches to assessment of entrepreneurs. In our research, we constructed a comprehensive trait framework by extracting insights from the practical experiences of accomplished startup founders. First, we systematically analyzed autobiographies of founders who built globally scaled companies across distinct geographic, institutional, and market contexts, including North America, Europe, Asia, and Australia: Elon Musk (SpaceX), Steve Jobs (Apple), Jeff Bezos (Amazon), Reid Hoffman (LinkedIn), Sara Blakely (Spanx), Hasso Plattner (SAP), Ingvar Kamprad (IKEA), Dilip Shanghvi (Sun Pharma), Jack Ma (Alibaba), Melanie Perkins (Canva). Once the set of founders was defined, we systematically examined each autobiography to identify characteristics that founders themselves consistently described as salient during their early life or early entrepreneurial phases, often predating venture success, and that they retrospectively linked to later company outcomes. This focus on early-emerging and persistent characteristics was intended to partially compensate for the absence of longitudinal data by prioritizing dispositions described as stable and formative rather than outcomes contingent on success.

Second, we conducted a total of 25 semi-structured interviews (60–90 minutes) with startup mentors, angel investors, and venture capitalists, predominantly from Silicon Valley and prominent European hubs. Collectively, these industry experts had over 250 years of founder-facing experience, and were selected based on track record, domain expertise, and ongoing hands-on support of successful entrepreneurs. Rather than beginning from theory, we aimed to surface practitioner-identified characteristics — those repeatedly observed by successful founders themselves and close observers-prioritizing the ones evident early and plausibly formative, as distinct from characteristics that may emerge only after success. These interviews were used solely to inform item generation and were not subjected to formal qualitative analysis or reported as empirical human-participant data.

Finally, we read financial articles and reports, from sources including Wealth-X and Hurun Research Institute, which focused on the characteristics of founders who built large-scale, high-growth ventures, and from platforms including Crunchbase and PitchBook that concentrate on startup ventures’ performance. These sources were used to supplement and cross-check insights derived from autobiographies and practitioner interviews, ensuring that candidate characteristics were not idiosyncratic to a single narrative source.

Characteristics that showed convergence across founders’ autobiographical accounts, practitioner interviews, and independent industry reports, particularly those described as early-emerging and persistent, were used to identify seven candidate dimensions: resilience, curiosity, value-creation/opportunism, courage, innovativeness, leadership, and emotional stability. These convergent themes were subsequently translated into candidate dispositional statements intended to reflect recurring founder-relevant behavioral tendencies, decision styles, and opportunity-oriented patterns described across sources. We then generated items intended to comprehensively represent these dimensions. This process initially yielded a pool of 60 items, which the authors subsequently evaluated for relevance, clarity, and redundancy, resulting in a refined pool of 49 items.

### 2.3. Face and content validity of the scale

The face and content validation procedures described below were conducted after initial item administration to the respondent sample, and were used to refine the item pool prior to psychometric modeling, not to alter or reinterpret participant responses.

The pool of 49 items was subjected to an external review process. To assess the content validity [59,60] of our instrument we engaged 10 experts (Domain Experts Panel), independent of the research group (as suggested by Roebianto et al. [61]), including venture capitalists, experienced angel investors, successful startup founders with double-digit million-dollar exits, and senior accelerator executives. Four of these individuals were based in the US, four in Europe, and two in Asia.

Following DeVellis’ recommendations [62], our Domain Expert Panel undertook a comprehensive evaluation, assessing each item’s (a) content relevance, (b) appropriateness, and (c) comprehensibility/clarity (as *per* Sireci & Faulkner-Bond [63]). To synthesize item scores (cf. McCoach et al. [64]), we employed the Item Content Validity Index (I-CVI) on a 1–4 scale (1 = *not relevant/appropriate/clear*, 2 = *need major revision*, 3 = *need minor revision*, 4 = *very relevant/appropriate/clear*). The I-CVI, calculated by the count of experts ratings of 3 or 4 to each item divided by the total experts, could range from 0 to 1 [65,66]. An I-CVI greater than.79 signifies items are relevant, between.70 and.79 items need revision, and below.70 should be removed [66]. The average Scale-level CVI (S-CVI/Ave) calculated by summing the I-CVIs and dividing by the total number of items, suggests an excellent content validity at ≥.9. Employing the same logic, we introduced Dimension-level CVI and computed D-CVI/Ave. Additionally, experts were consulted on new dimensions, items, and wording improvements.

To establish Face validity [66], a second external panel, the Lay Experts Panel [65], consisting of 10 individuals representing likely questionnaire respondents (e.g., early-stage startup founders and graduate students interested in entrepreneurship), provided similar feedback as the Domain Expert Panel (S1 Table) for each of the proposed items. The Lay Experts Panel did not evaluate whether the dimensions reflected relevant success characteristics for startup founders.

Out of 49 items, 47 (as *per* the Domain Expert Panel) and 46 (*per* the Lay Expert Panel) demonstrated high content validity (CVI values ≥ .80). Similarly, 48 (Domain Expert Panel) and 47 (Lay Expert Panel) items exhibited high appropriateness, while 47 (Domain Expert Panel) and all 49 (Lay Expert Panel) items were deemed clear and comprehensive (S1 Table). However, unanimously, two items from Emotional Stability, and one item from Leadership domain were considered not relevant, appropriate, or clear (CVI values ≤ .70) and were subsequently eliminated. This resulted in 46 remaining items.

Further, we assessed whether the Domain Expert Panel considered each proposed dimension essential for capturing the instrument’s targeted construct [67,68]. Using the Content Validity Ratio (CVR) method, adjusted for dimensions on a 1–3 scale (1 = *not necessary*, 2 = *useful but not essential*, 3 = *essential*), CVR was calculated as: CVR = (Ne − N/2)/ (N/2), where Ne is the number of experts rating the dimension as ‘essential’ and N is the total number of experts.

Considering the critical value for acceptance of >.62 for 10 experts [67], the Emotional Stability dimension having a CVR value of.40 (see Table 2), and relatively lower CVI-I and CVI-D/Ave scores for relevance, appropriateness, and clarity, was removed. This led to a refined set of 41 items distributed across the remaining six dimensions.

**Table 2:** Domain experts panel assessments of content validity ratio (CVR) for dimensions.

Dimensions	Curiosity	Innovative ness	Resilience	Emotional Stability	Value-creation/ Opportunism	Leadership	Courage
CVR	.80	.80	1.00	**.40**	.80	1.00	.80

Dimensions with CVR values in bold fell below the accepted cut-off, suggesting less consensus on their criticality, and were considered for elimination from further analysis.

### 2.4. Item format and administration

Items were rated on a five-point Likert scale ranging from 1 (“agree least”) to 5 (“agree most”). The initial item pool was administered online using a secure survey platform. Items were presented in randomized order with respect to underlying dimensions to reduce potential order and clustering effects, while maintaining a fixed order within each participant. All items were mandatory, resulting in no missing item-level data.

Although 49 items were administered to the respondent sample, only the 41 items retained following panel review were entered into the EFA.

### 2.5. Method selection and suitability

Given the goals and exploratory aims of this study and the absence of a predefined factor structure, an exploratory factor analysis (EFA) was selected to identify the latent dimensions underlying the item set without imposing a priori assumptions or constraints. Sampling adequacy was assessed using the Kaiser–Meyer–Olkin (KMO) measure of sampling adequacy (MSA; [69]), with 30 items demonstrating values between.90 and 1.00 (‘marvelous’) and 10 items falling between.80 and.90 (‘meritorious’) according to established criteria [70]. The overall KMO MSA value was.930, indicating excellent sampling adequacy.

Bartlett’s test of sphericity was significant (χ²(820) = 150,220.93, p < .0001), indicating that the item correlation matrix was suitable for exploratory factor analysis.

#### 2.5.1. Exploratory factor analysis (EFA).

Exploratory factor analyses were conducted in *R* (version 4.2.2; GUI 1.79; Big Sur ARM build) using *principal axis factoring (PAF)* with *Promax* rotation, allowing for correlated latent factors. EFA was conducted twice following identical procedures: first on a randomly selected half of the total sample across all participant groups, and second on the full sample of Successful Startup Founders (see S2 Table). This dual-sample approach was used to assess the stability of the factor structure both in the heterogeneous population and within the focal group of successful founders.

PAF was selected because it estimates shared variance among items [71,72], relies on fewer distributional assumptions than maximum likelihood estimation [73], and provides more accurate solutions than principal component analysis when communalities are modest [74]. An oblique rotation (Promax with Kaiser normalization) was applied in line with recommendations for psychological constructs expected to be intercorrelated [75,76].

Items with communalities below.40 were removed. Retained items were required to load ≥ .50 on their primary factor [77,78], exhibit cross-loadings ≤ .32 [76], and demonstrate a minimum loading difference of.20 between primary and secondary factors [73].

As an additional diagnostic check, the raw Pearson correlation matrix was inspected to verify that inter-item relationships were consistent with the extracted factor structure.

#### 2.5.2. Deciding on the number of model factors.

The number of factors to retain was determined using multiple complementary criteria [76,79]. Kaiser’s eigenvalue-greater-than-one rule [80], which is known to over-extract factors [81,82], was used as an initial heuristic and supplemented with parallel analysis [83] to provide a more precise estimate [84]. Parallel analysis was implemented following Dinno’s approach [85], which is applicable to non-normal data distributions [86], by comparing observed eigenvalues to those generated from random datasets. As suggested by Montanelli and Humphreys [87], the point of intersection between observed and random eigenvalues was used to identify factors exceeding variance expected by chance. In addition, the break point was evaluated by plotting the smallest observed eigenvalues [88]. Convergence across these criteria was further evaluated using Cattell’s scree test [89], which, despite some subjectivity, is considered an intuitive and generally accurate decision aid [90].

#### 2.5.3. Confirmatory factor analysis (CFA).

Confirmatory factor analyses were conducted to evaluate the fit of the derived factor structure. CFA was performed in *R* (version 4.2.2; GUI 1.79; Big Sur ARM build) using the *psych* and *lavaan* packages, with results cross-verified in *Jamovi* (version 2.3.21.0). Given the large sample size and the five-point *Likert* response format, items were treated as approximately continuous indicators and models were estimated using maximum likelihood (ML) estimation, with factor variances fixed to 1 for model identification. Given the large sample size and approximately normal item distributions, ML estimation was deemed appropriate and robust.

Following recommendations by Willmer et al. [91], CFA was conducted on the remaining half of the total sample not used for EFA and was evaluated separately for each participant group (Successful Startup Founders, Corporate Managers, and Aspiring Entrepreneurs). Model fit was assessed using the Comparative Fit Index (CFI), Tucker–Lewis Index (TLI), Root Mean Square Error of Approximation (RMSEA), Standardized Root Mean Square Residual (SRMR), Akaike Information Criterion (AIC), and Bayesian Information Criterion (BIC). Both five-factor and six-factor solutions were tested, consistent with results from parallel analysis, to assess model robustness across groups.

Additionally, multi-group measurement invariance analyses were conducted across Successful Startup Founders (SSF), Corporate Managers (CM), and Aspiring Entrepreneurs (AE) using the final six-factor model. A hierarchical sequence of configural, metric, scalar, and strict invariance models was evaluated to examine the stability of the latent structure across groups [92,93]. Given the large sample size, practical fit indices (CFI, TLI, RMSEA, SRMR) were prioritised over chi-square difference significance tests when evaluating invariance.

#### 2.5.4. Examining factors relationship, convergent and discriminant validity, and scale reliability.

Following the EFA and CFA, interrelationships between latent factors were examined using a between-factor correlation matrix. Convergent validity was assessed by evaluating the unidimensionality of each latent factor [58,94,95], while discriminant validity was examined by evaluating the distinctiveness of factors. Specifically, the Average Variance Extracted (AVE) was calculated for each latent factor as the mean of the squared standardized factor loadings relative to the total variance accounted for by both the loadings and the corresponding error variances [96]. Discriminant validity was evaluated by comparing the square root of AVE for each factor with the inter-factor correlations, with square root AVE values exceeding the corresponding correlations indicating adequate discriminant validity.

Scale reliability was assessed using both Cronbach’s alpha [97] and McDonald’s omega [98] to evaluate internal consistency and robustness of the latent constructs.

#### 2.5.5. Exploring data distributions and normality.

To examine the underlying data structure, mean factor scores were visually inspected across respondents. Violin box plots were used to illustrate score distributions across the three participant groups. Normality was further evaluated using Q–Q plots, which are appropriate for large samples [99], alongside the Kolmogorov–Smirnov test and skewness and kurtosis statistics to identify deviations from normality across factors and groups.

### 2.6. Evaluating between-group differences

#### 2.6.1. Robust tests for equality of means and post-hoc tests.

Given the large sample size and the known sensitivity of formal normality tests under such conditions [100], robust tests for equality of means were employed. Welch’s [101] and Brown–Forsythe [102] tests were used to accommodate non-homogeneous variances, consistent with recommendations by Lix et al. [103] and Clinch and Keselman [104]. Post-hoc comparisons were conducted using Tamhane’s T2 and Games–Howell tests, which are robust to unequal variances and unequal group sizes [105].

Effect sizes were quantified using Cohen’s d [55] with pooled standard deviations, calculated as *d* = (M₁ – M₂)/ SD_pooled, where SD_pooled = √[((n₁ – 1)SD₁² + (n₂ – 1)SD₂²)/ (n₁ + n₂ – 2)].

#### 2.6.2. Regression factor score analysis.

To examine differences in latent factor expression across participant groups, regression-based factor score estimation was employed [106]. Factor scores were standardized to a mean of zero and scaled according to the squared multiple correlation between observed variables and latent factors. Unlike simple summed scores, this approach estimates factor scores using regression weights derived from item loadings and factor correlations, accounting for both magnitude and directionality of associations. Positive factor scores indicate above-average association with the corresponding latent factor, whereas negative scores indicate below-average association.

## 3. Results

### 3.1. Model development and evaluation

#### 3.1.1. Determination of the number of factors.

Kaiser’s Eigenvalues suggested six factors, whereas Parallel Analysis indicated five (see Table 3). Both methods indicated that the extracted factors explained variance exceeding that expected by chance.

**Table 3:** Results from Kaiser’s rule and parallel analysis (PA) for factor retention.

Factors	Eigenvalues Observed from EFA	Simulated/Random Eigenvalues from PA	Retained Factors
Factor 1	**20.61**	**2.42**	Accept
Factor 2	**5.24**	**2.21**	Accept
Factor 3	**3.60**	**2.20**	Accept
Factor 4	**3.14**	**2.03**	Accept
Factor 5	**2.85**	**2.00**	Accept
Factor 6	**1.41**	1.79	Consider
Factor 7	0.78	1.52	Reject

Factors meeting both criteria are labelled ‘Accept,’ with values surpassing the recommended cut-off in bold. Factor 6 is labeled ‘Consider’ due to diverging results between Parallel Analysis and Kaiser’s criterion. Factor 7 is marked as ‘Reject’ by both analyses.

**Fig 1:**
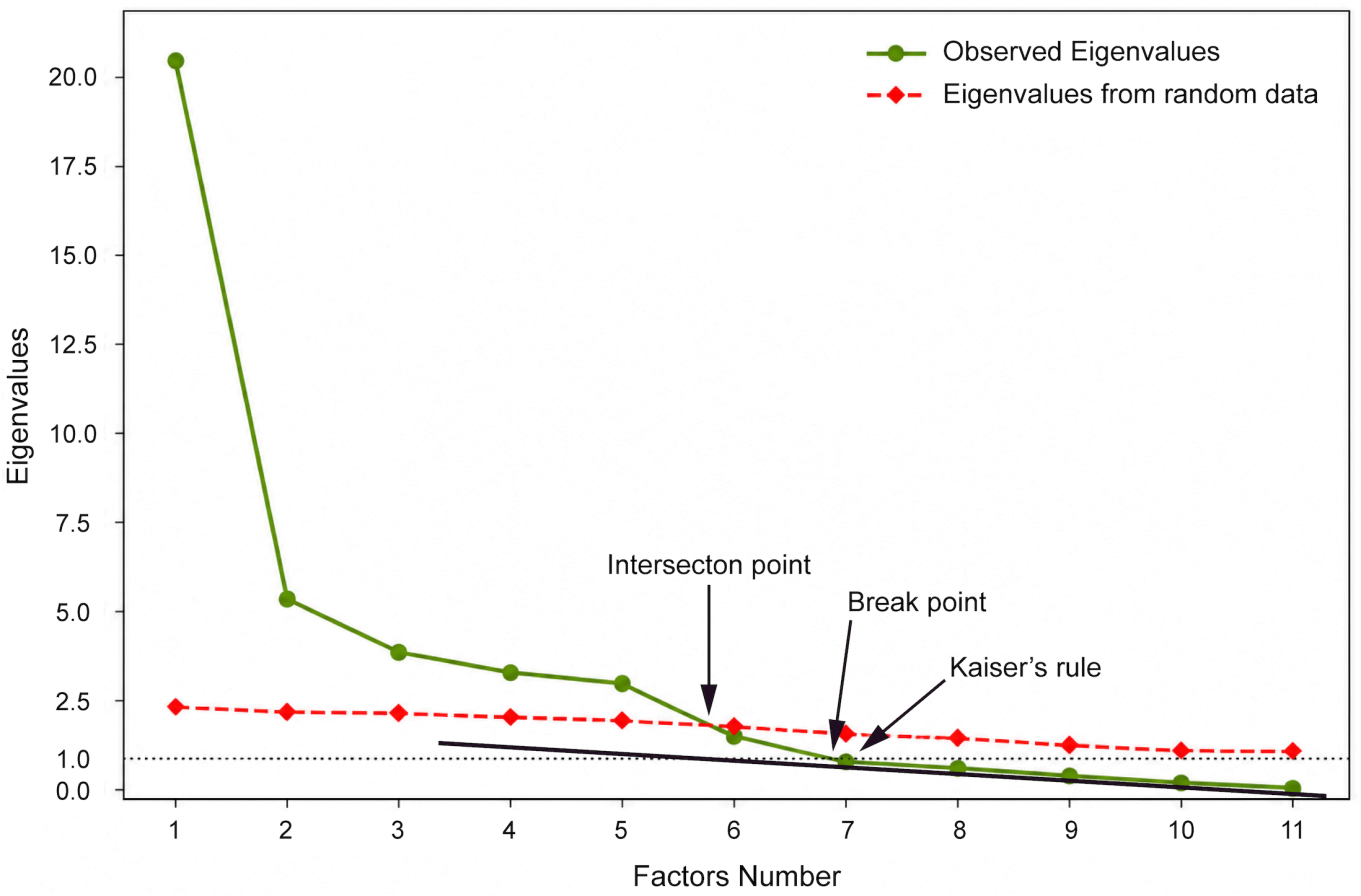
Scree plot with observed and random eigenvalues. The green solid line represents the observed eigenvalues, while the red dashed line represents eigenvalues generated from random data in the parallel analysis. Factors with eigenvalues greater than 1 [80], factors above the scree ‘elbow’ or ‘break point’ [88], and factors above the intersection between observed and random eigenvalues in the parallel analysis [87] may be considered for factor retention.

A visual inspection of the scree plot (Fig 1) suggested the presence of six factors based on both the distinct ‘elbow’ and Kaiser’s rule [107]. However, the intersection of eigenvalues from observed and random data indicated that only 5 factors met this criterion. Consequently, we opted to consider both a five- and a six-factor model for further analysis.

#### 3.1.2. Results of the exploratory factor analysis (EFA).

Following the described methodology and beginning with the 41 items retained after panel review, the EFA was conducted over four iterative rounds, resulting in a final factor structure comprising 31 items. These items exhibited substantial loadings, ranging from.518 to.854. Only one item exhibited a cross-loading within the range of.30 to.32 but had a primary factor loading of >.50, with a loading difference of >.20 from its alternative factor. Consequently, the item was retained given its strong primary loading and clear separation from the secondary factor (see Table 4). The between-item Pearson’s *R* correlation matrix is illustrated in S1 Fig.

**Table 4:** Item-to-latent factor correlations.

Items	Factors					
	1	2	3	4	5	6
Item 15	**.854**	−.040	−.110	.017	.042	.042
Item 16	**.833**	−.040	.009	−.019	.051	.032
Item 18	**.748**	−.091	.036	.001	.050	−.107
Item 17	**.742**	.127	.035	−.060	−.035	−.022
Item 19	**.689**	.099	−.067	.037	.003	−.020
Item 20	**.610**	−.040	.161	−.001	−.066	.170
Item 38	−.003	**.827**	.028	−.121	.062	−.019
Item 36	−.020	**.782**	.015	−.092	−.009	.060
Item 40	−.012	**.736**	.050	−.054	.099	−.014
Item 39	.039	**.715**	.020	.031	.030	.036
Item 41	−.029	**.567**	.194	.081	.076	−.032
Item 22	−.100	.069	**.756**	−.035	−.131	.029
Item 23	.072	−.031	**.751**	.084	−.033	−.057
Item 24	−.006	−.037	**.627**	.172	.123	.028
Item 26	.057	.038	**.626**	.039	−.032	.036
Item 25	.040	.305	**.583**	.014	−.123	−.032
Item 2	−.010	−.065	−.077	**.775**	.009	−.071
Item 1	−.047	−.070	.053	**.733**	.000	.092
Item 6	.031	.024	.095	**.599**	−.022	−.109
Item 4	−.045	−.037	.184	**.594**	.096	−.006
Item 5	.062	−.113	.107	**.566**	.166	.043
Item 33	−.003	.169	−.104	.128	**.710**	−.026
Item 31	.011	.216	−.176	.074	**.709**	−.068
Item 30	−.060	−.090	.029	.026	**.684**	.140
Item 32	.252	−.064	.100	−.169	**.586**	−.095
Item 35	−.049	.102	−.120	.123	**.518**	.108
Item 8	−.014	.045	.060	−.039	−.022	**.733**
Item 9	.026	.114	−.139	.151	−.102	**.724**
Item 10	−.119	−.127	.194	−.257	.250	**.696**
Item 12	.043	−.109	.038	−.092	.181	**.655**
Item 11	.123	.163	−.183	.219	−.240	**.572**

Factor loadings exceeding.50 are highlighted in bold, and a cross-loading falling between.30 and.32 is underlined.

The factor loadings from the EFA of the Successful Startup Founders (see S2 Table), shows high values across most items, indicating these characteristics are strongly defined among successful founders. Despite similarly high loadings observed in the EFA encompassing all three groups (Table 4), the values appear slightly more diluted. This pattern indicates that although these characteristics are present across groups, factor loadings were strongest among successful founders.

#### 3.1.3. Confirmatory factor analysis (CFA) outcomes.

Confirmatory factor analyses tested both five- and six-factor solutions across Successful Startup Founders (SSF), Corporate Managers (CM), and Aspiring Entrepreneurs (AE) (Table 5). Across all groups, model fit was strongest for the SSF sample. Within SSF, the five-factor model demonstrated superior fit across all indices (CFI = .984, RMSEA = .037), whereas the six-factor model also met conventional thresholds for acceptable fit, albeit with modestly reduced indices (CFI = .909, RMSEA = .070).

**Table 5:** CFA with successful startup founders, corporate managers, and aspiring entrepreneurs: five- versus six-factor models.

Number of factors	Group	CFI	TLI	SRMR	RMSEA	AIC	BIC
5 factors (without TRL)	**Successful Startup Founders**	**.984**	**.977**	**.0326**	**.0368**	**12224**	**12430**
	Corporate Managers	.877	.861	.0455	.0614	57360	57432
	Aspiring Entrepreneurs	.757	.720	.0703	.1050	86967	87410
6 factors (including TRL)	**Successful Startup Founders**	**.909**	**.881**	**.0469**	**.0702**	**58155**	**58459**
	Corporate Managers	.849	.830	.0498	.0835	66996	67587
	Aspiring Entrepreneurs	.719	.683	.0710	.0910	98812	99305

Higher CFI/TLI and lower SRMR/RMSEA/AIC/BIC indicate better fit. Best-fitting group within each model is bolded.

In the CM and AE groups, both models exhibited lower overall fit, with the five-factor solution consistently outperforming the six-factor model. This pattern indicates that while the five-factor structure represents a more parsimonious and statistically robust solution across heterogeneous groups, the six-factor model remains structurally adequate, particularly within the focal group of successful founders.

3.1.3.1. ***Measurement Invariance Across Entrepreneurial and Comparison Groups***. To examine the stability of the SFSS measurement structure across different entrepreneurial and professional populations, multi-group confirmatory factor analyses were conducted across Successful Startup Founders (SSF), Corporate Managers (CM), and Aspiring Entrepreneurs (AE) using the final six-factor, 31-item model.

A sequence of increasingly constrained invariance models was evaluated, including configural, metric, scalar, and strict invariance models (Table 6). The configural model demonstrated good overall fit, supporting the presence of the same six-factor structure across all three groups (CFI = .952, TLI = .947, RMSEA = .046, SRMR = .049).

**Table 6:** Multi-group measurement invariance analyses across successful startup founders (SSF), corporate managers (CM), and aspiring entrepreneurs (AE).

Model	χ²	df	CFI	TLI	RMSEA	SRMR
Configural	10077.93	1257	.952	.947	.046	.049
Metric	10159.24	1307	.952	.948	.045	.049
Scalar	11214.08	1357	.946	.945	.047	.054
Strict	12734.88	1419	.938	.939	.049	.055

Given the large overall sample size (N = 10,007), practical fit indices were prioritised over chi-square significance tests in evaluating invariance.

Constraining factor loadings to equality across groups resulted in minimal changes in practical fit indices (metric invariance: CFI = .952, RMSEA = .045, SRMR = .049), supporting substantial metric invariance across groups despite significant chi-square difference tests. Additional scalar constraints produced modest reductions in model fit (scalar invariance: CFI = .946, RMSEA = .047, SRMR = .054), while the strict invariance model showed further but still acceptable deterioration (strict invariance: CFI = .938, RMSEA = .049, SRMR = .055).

These findings are consistent with the earlier group-specific CFA results, in which model fit was strongest within the SSF sample and comparatively weaker within the CM and AE groups. Overall, the results support the stability of the SFSS latent structure across entrepreneurial and comparison populations, while also indicating the presence of some group-specific variation in item intercepts and residual variances.

### 3.2. Interpreting the extracted factors

Factor 1: Relentless Resilience (RER) refers to an entrepreneur’s sustained ability to bounce back from setbacks and adversity, with unwavering determination amid diverse and ongoing challenges and risks inherent to the entrepreneurial role. It encompasses the capacity to persist through difficulties, delay gratification when necessary, learn, adapt, and grow both personally and professionally. RER accounted for 30.92% of the common variance. Representative items included statements such as “I am willing to try again and again when I experience setbacks.” Factor 2: Value-Creating Opportunism (VCO) encompasses an entrepreneur’s inclination and ability to promptly recognize and seize less evident but exceptionally promising emerging opportunities, in terms of both potential for financial growth and value creation while addressing real-world challenges. It integrates strategic vision, risk management, and resourcefulness. VCO accounted for 10.10% of the common variance. Example items included “I know that most bad things in the world can become business opportunities.” Factor 3: Intrinsic Curiosity (INC) includes entrepreneurs’ inclination to expand their knowledge, explore uncharted territories, and envision connections between diverse fields, fostering groundbreaking ideas and solutions to reshape industries and create new markets. INC accounted for 9.35% of the common variance. Illustrative items included “I am curious to learn and explore subjects unrelated to my expertise.” Factor 4: Courageous Decision-Making (CDM) encapsulates the fortitude of entrepreneurs to navigate business politics and competition, and to act decisively under pressure and ambiguity. It involves mental toughness and the embrace of discomfort, while prioritizing authenticity over approval. CDM accounted for 8.09% of the common variance. Representative items included “I am able to do what I should do even when I feel scared.” Factor 5: Strategic Innovativeness (STI) captures an entrepreneur’s ability to strategically transform creative ideas into practical and groundbreaking solutions that strongly resonate with customers and markets. It reflects that entrepreneurs don’t innovate solely for the sake of innovation; instead, they focus on areas that align with their financial objectives and overarching vision. STI accounted for 6.93% of the common variance. Example items included “I am able to see the big picture and innovate with strategic purpose.” Factor 6: Transformational Leadership (TRL) encompasses the capacity to empower and influence individuals to unite around a shared vision, confidently leading through uncharted territories, and navigating uncertainty and risks while guiding their companies forward. TRL accounted for 5.55% of the common variance. Representative statements included “I prefer to take on the leadership role in a group to empower others.”

### 3.3. Variance explained by the scale

Our six-factor model, comprised of 31 items, revealed substantial explanatory power, accounting for approximately 71% of the common variance among the retained items across all three groups (see Table 7).

**Table 7:** Percentage of common variance explained by the six-factor solution (31 Items).

Factors	Extraction Sums of Squared Loadings	
	% of Variance	Cumulative %
Relentless Resilience (RER) (Items 15, 16, 17, 18, 19, 20)	30.92	30.92
Value-Creating Opportunism (VCO) (Items 36, 38, 39, 40, 41)	10.10	41.02
Intrinsic Curiosity (INC) (Items 22, 23, 24, 25, 26)	9.35	50.37
Courageous Decision-Making (CDM) (Items 1, 2, 4, 5, 6)	8.09	58.45
Strategic Innovativeness (STI) (Items 30, 31, 32, 33, 35)	6.93	65.38
Transformational Leadership (TRL) (Items 8, 9, 10, 11, 12)	5.55	**70.94**

The table displays the percentage of common variance explained by each extracted factor and the cumulative percentage of common variance explained across factors. Values are based on the Extraction Sums of Squared Loadings from the principal axis factoring (PAF) solution.

### 3.4. Factor relationship, convergent and discriminant validity, and scale reliability

The most prominent correlation was observed between Relentless Resilience (RER) and Strategic Innovativeness (STI) at.513 (see Table 8), well below the thresholds suggested to avoid discriminant validity issues at.80 or.85 [95,108] and Shao et al.‘s even more conservative.70 cutoff [109] to prevent collinearity. This correlation indicates that higher levels of STI tend to co-occur with higher levels of RER. Overall, our findings demonstrate adequate convergent validity, meeting Götz et al.’s cutoff criteria (AVE ≥ .50) [110]. We also confirmed discriminant validity in our model as per Malhotra’s [96] guideline, which requires the square root of the Average Variance Extracted (√AVE) to exceed between-factor correlation coefficients (see Table 9).

**Table 8:** Between-factor correlations.

Factors	RER	VCO	INC	CDM	STI	TRL
Relentless Resilience (RER)	1.000					
Value-Creating Opportunism (VCO)	.420	1.000				
Intrinsic Curiosity (INC)	.403	.433	1.000			
Courageous Decision-Making (CDM)	.469	.464	.459	1.000		
Strategic Innovativeness (STI)	.513	.425	.351	.339	1.000	
Transformational Leadership (TRL)	.299	.253	.124	.247	.248	1.000

**Table 9:** Discriminant validity assessment, Cronbach’s alpha, and McDonald’s omega.

Factors	√AVE	Discriminant Validity	Cronbach’s Alpha	McDonald’s Omega
Relentless Resilience (RER)	.751	√AVE₁ > all correlations	.861	.859
Value-Creating Opportunism (VCO)	.731	√AVE₂ > all correlations	.808	.808
Intrinsic Curiosity (INC)	.673	√AVE₃ > all correlations	.818	.818
Courageous Decision-Making (CDM)	.659	√AVE₄ > all correlations	.739	.737
Strategic Innovativeness (STI)	.646	√AVE₅ > all correlations	.746	.744
Transformational Leadership (TRL)	.678	√AVE₆ > all correlations	.712	.719

Examination at the item level consistently revealed high Omega and Alpha values > .70, with ‘Relentless Resilience’ being the highest, and ‘Transformational Leadership’ the lowest, as also reflected in the corresponding McDonald’s Omega values (see Table 9), with no improvement in results per factor through the deletion of specific items. These assessments collectively suggest the robustness of the scale structure, ensuring that the items within each factor consistently measure the same underlying construct.

### 3.5. Between-groups comparisons and differences

**Fig 2:**
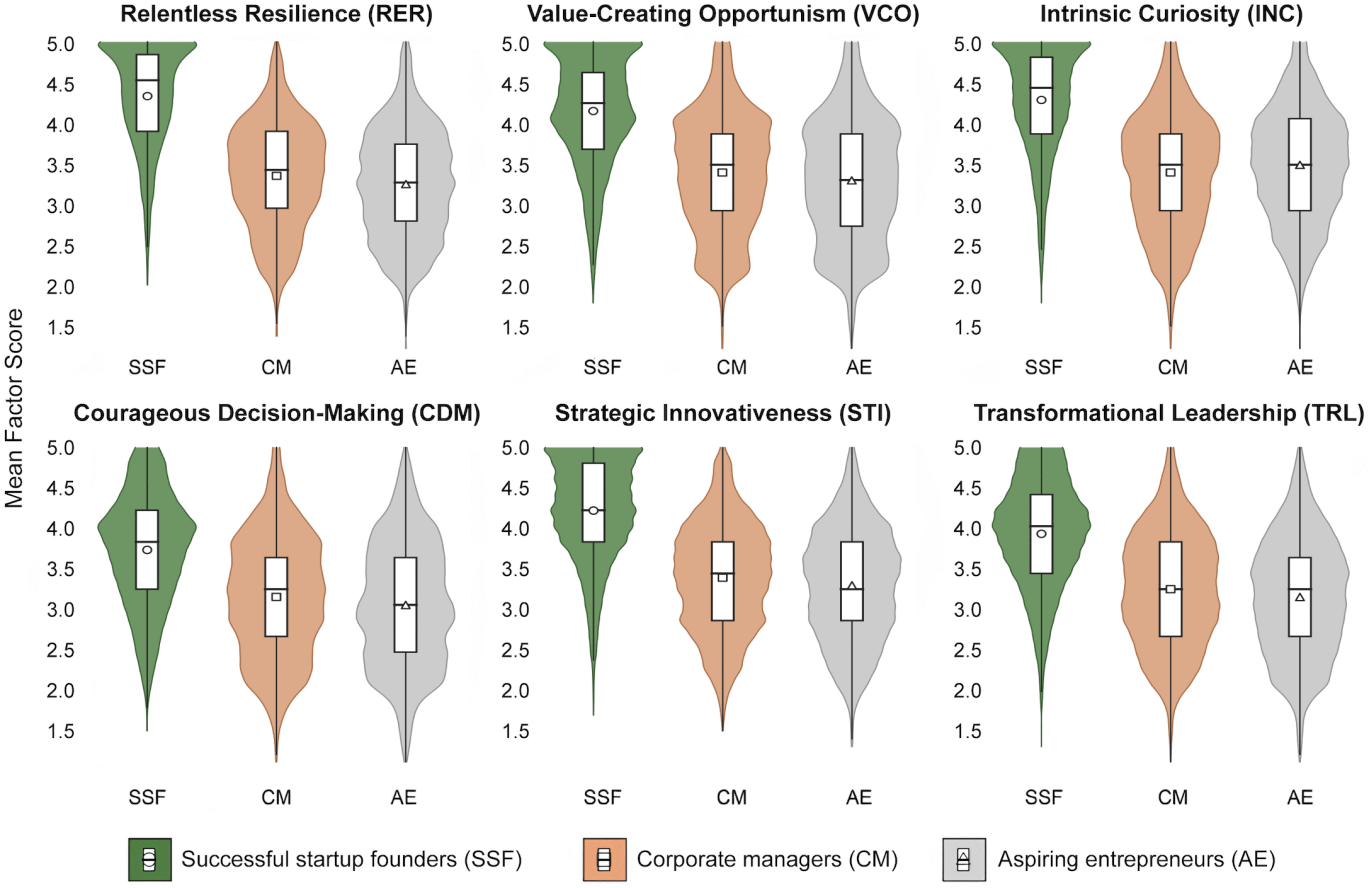
Violin box plots of mean factor score distributions across participant groups. The violin plots illustrate the distribution of mean factor scores for Successful Startup Founders (SSF), Corporate Managers (CM), and Aspiring Entrepreneurs (AE) across six found characteristics. The central horizontal line within each box indicates the median, while circle, square, and triangle markers represent the mean values for SSF, CM, and AE, respectively.

We compared the mean factor scores among the three different participant groups (see Fig 2), supplemented by Q–Q plots and Kolmogorov-Smirnov tests (see S2 Fig and S3 Table, respectively).

Robust one-way tests (Welch and Brown–Forsythe) indicated significant group differences among Successful Startup Founders (SSF), Corporate Managers (CM), and Aspiring Entrepreneurs (AE) across all six SFSS factors (all *p* < .001; Table 10). See S4 Table for skewness and kurtosis.

**Table 10:** Robust tests of mean differences across groups for SFSS factors.

Dependent Variable	Robust tests of equality of means	*F*	df1	df2	*p*
Relentless Resilience (RER)	Welch	25.470	2	3707.576	<.001
	Brown-Forsythe	28.120	2	5358.784	<.001
Value-Creating Opportunism (VCO)	Welch	35.157	2	3796.879	<.001
	Brown-Forsythe	34.927	2	5640.805	<.001
Intrinsic Curiosity (INC)	Welch	46.169	2	3740.941	<.001
	Brown-Forsythe	45.466	2	5577.785	<.001
Courageous Decision-Making (CDM)	Welch	30.665	2	3640.862	<.001
	Brown-Forsythe	33.038	2	5204.598	<.001
Strategic Innovativeness (STI)	Welch	125.797	2	3757.007	<.001
	Brown-Forsythe	133.794	2	5508.554	<.001
Transformational Leadership (TRL)	Welch	84.589	2	3717.776	<.001
	Brown-Forsythe	96.520	2	5255.758	<.001

Welch’s and Brown–Forsythe robust tests were used due to unequal variances and unequal group sizes. The distribution is asymptotically F. Tests were conducted on regression factor scores derived from the EFA solution.

Post-hoc Games–Howell comparisons and descriptive mean scores analyses (see S5 Table and S3 Fig, respectively), revealed a consistent pattern. On regression factor scores, SSF and CM showed similar levels of RER, with both exceeding AE; SSF scored higher than both CM and AE on VCO; with no significant differences between CM and AE; SSF also displayed consistently higher levels of CDM, STI, and TRL than both CM and AE, with CM scoring higher than AE on each. Interestingly, while SSF scored highest on INC, AE outscored CM on both regression factor scores and descriptive mean scores, marking it as the sole dimension where AE surpassed CM, though this difference was not statistically significant. Descriptive mean scores (S3 Fig and Table 11) confirmed this overall pattern, with SSF consistently scoring highest across all dimensions. This profile underscores that success-linked entrepreneurial characteristics are most pronounced in SSF, partially present in CM, and generally weakest in AE.

Effect sizes (Cohen’s d) were calculated using pooled standard deviations. SSF vs. CM comparisons yielded d ranging from 0.83 to 1.62; SSF vs. AE from 0.97 to 1.77 — all classified as large (d > 0.8 [55]). The strongest separations were in Relentless Resilience (d = 1.62 vs. CM; 1.77 vs. AE) and Strategic Innovativeness (d = 1.27 vs. CM; 1.42 vs. AE). See Table 11 for full descriptive mean scores, standard deviations, and corresponding effect sizes.

**Table 11:** Group means, standard deviations, and effect sizes (Cohen’s d) on SFSS dimensions.

Dimension	SSF M (SD)	CM M (SD)	AE M (SD)	d (SSF vs CM)	d (SSF vs AE)
Relentless Resilience (RER)	4.30 (.65)	3.25 (.70)	3.15 (.72)	1.62	1.77
Value-Creating Opportunism (VCO)	4.10 (.70)	3.30 (.75)	3.20 (.77)	1.14	1.29
Intrinsic Curiosity (INC)	4.25 (.68)	3.30 (.75)	3.40 (.73)	1.40	1.25
Courageous Decision-Making (CDM)	3.70 (.72)	3.10 (.77)	3.00 (.79)	.83	.97
Strategic Innovativeness (STI)	4.20 (.67)	3.35 (.72)	3.25 (.74)	1.27	1.42
Transformational Leadership (TRL)	3.90 (.70)	3.20 (.75)	3.10 (.77)	1.00	1.14

Cohen’s d calculated using pooled SD. All d > 0.8 indicate large effect sizes [55].

**Fig 3:**
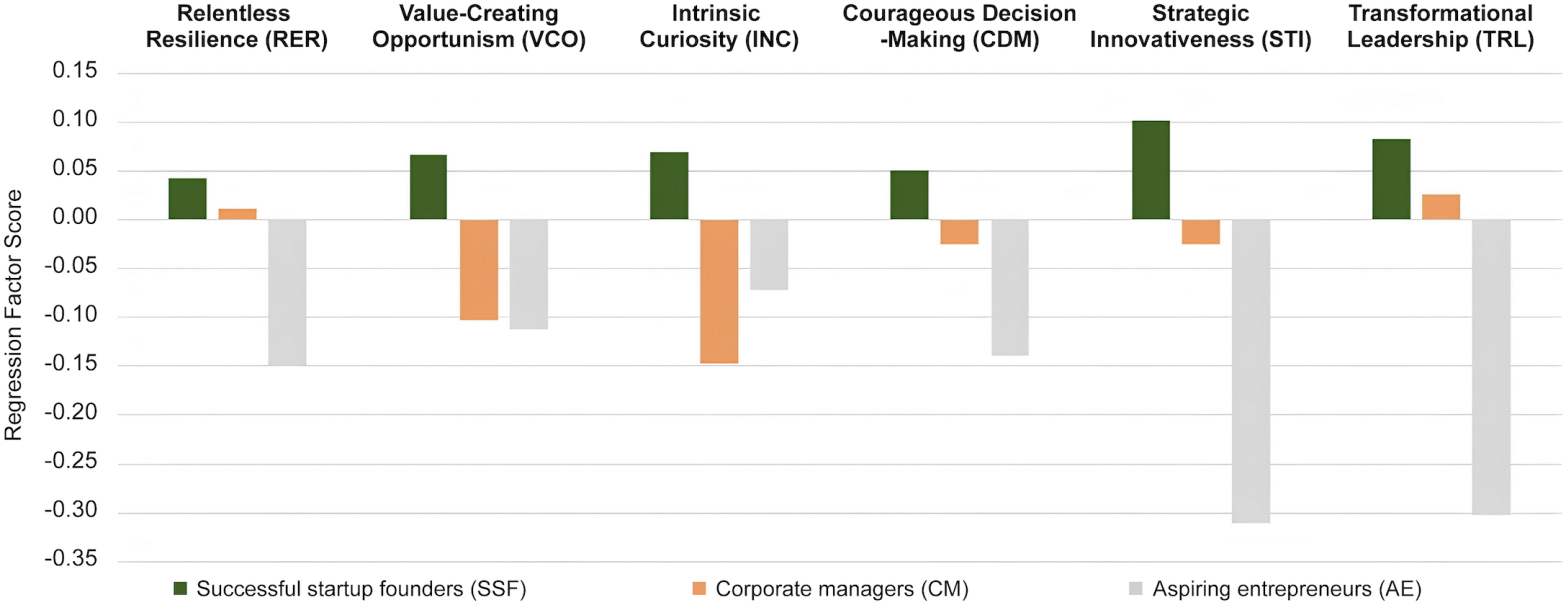
Mean regression factor scores across participant groups and founder characteristics. Positive factor scores indicate above-average association with the corresponding latent factor, whereas negative factor scores indicate below-average association.

Furthermore, our Regression Factor Score Analysis (Fig 3) shows the relative differences and directionality in these associations among the factors scores for the six founder characteristics across our three participant groups. SSF tended to show stronger positive associations with all six factors, while CM and AE show varying degrees of comparatively weaker associations with some of these factors. Notably, AE exhibit much weaker relationships with RER, STI, and TRL compared to the other two groups. Whilst CM show some positive relationships with RER and TRL.

## 4. Discussion

### 4.1. Founders characteristics underlying startup success

Guided by founder narratives and expert interviews, our EFA/CFA approach isolated six characteristics that consistently co-occur among successful startup founders and together account for a large proportion of variance in founder-specific trait measures, reflecting strong internal coherence of the proposed construct. This level of construct coverage contrasts with prior work showing that broad personality traits tend to exhibit only modest associations with entrepreneurial outcomes [16,17]. In addition, between-group comparisons revealed large effect-size separations between Successful Startup Founders and both Corporate Managers and Aspiring Entrepreneurs (Cohen’s d = 0.83–1.77), indicating that these characteristics are most pronounced among successful founders. These effects are substantially larger than the small-to-moderate effect sizes (typically d ≈ 0.2–0.5) reported when broad personality traits are related to entrepreneurial outcomes in prior research [17,18,51].

Confirmatory factor analysis demonstrated strong model fit among Successful Startup Founders and acceptable fit in comparison groups, supporting the structural robustness of the construct, particularly within the Successful Startup Founder group. Taken together, these findings support the SFSS as a rigorously derived, founder-specific model of success characteristics, with potential relevance for research, investor screening, accelerator assessment, and entrepreneurship education.

Conceptually, our findings support a multi-level conceptual framework in which founder characteristics are likely to influence (i) decision policies under uncertainty (e.g., thresholds for action, loss tolerance, willingness to act with incomplete information), and (ii) resource orchestration (e.g., opportunity selection, sequencing of capital, mobilizing talent and partners). These behavioral pathways appear to be associated with differences in early execution patterns and outcomes, which may relate to investor interest and subsequent scaling processes. This framework complements intent-focused models by clarifying *how* founder-specific characteristics may relate to decision-making under uncertainty and venture-level outcomes in ways not fully captured by broad personality measures.

### 4.2. Differences and similarities with prior ‘one-trait’ models

Each of our six dimensions builds upon and clarifies prior one-trait models in the entrepreneurial context: relentless resilience (RER) with persistence and adversity recovery, value-creating opportunism (VCO) with opportunity recognition or creation for maximum expected financial value and scalable impact, intrinsic curiosity (INC) with knowledge-seeking, cross-domain connections and breakthrough insight generation, courageous decision-making (CDM) with fortitude under uncertainty and calculated discomfort, strategic innovativeness (STI) with targeted, financially aligned novelty, and transformational leadership (TRL) with visioning, empowerment, and influence.

Our model therefore offers a comprehensive, multi-factor account of successful founders that distinguishes itself from prior ‘one-trait’ models (e.g., [25–36]. Below, we evaluate each characteristic in comparison to the existing literature.

*Relentless Resilience (RER)*: Resilience is widely acknowledged as essential for entrepreneurial success [34,111]. Yet, definitions vary [112,113] from hardiness and persistence [111] vs. resourcefulness and optimism [34], among others. In our model, RER incorporates persistence, recovery from adversity [114], and willingness to delay rewards [115]. We term it ‘Relentless’ because successful founders must sustain this capacity across repeated, diverse challenges—preserving capital and execution momentum to enable bounce-back and exit readiness.

*Value-Creating Opportunism (VCO)*:The pursuit of opportunities is a core driver of entrepreneurship [116,117] wealth creation and success [118,119]. Whether opportunities are discovered through intuition, alertness [120], and pattern recognition, or created with innovation and resource reconfiguration [121–123], prior work often emphasizes opportunity recognition and exploitation while underplaying the central role of expected value creation (e.g., [124–126]. Our findings emphasize that successful founders systematically prioritize opportunities based on maximum expected financial value and scalable impact, whether identified or created. High VCO may support capital-efficient growth and stronger exit positioning.

*Intrinsic Curiosity (INC)*: Previous studies often subsume curiosity under the Big Five’s Openness to Experience (e.g., [127]), a broad construct spanning vivid imagination to aesthetic sensitivity. In contrast, we define INC as the intrinsic drive to seek and acquire new knowledge [128–130], forge cross-domain connections, and generate breakthrough insights, consistent with evidence linking curiosity to firm growth via innovation [131]. High INC may contribute to first-mover advantages and defensible intellectual property, supporting rapid market capture and long-term scalability.

*Courageous Decision-Making (CDM)*: Courage has been tied to increased entrepreneurial activity [132] and potentially entrepreneurial success (e.g., [133,134]), though often measured subjectively via entrepreneurs’ Psychological Capital (PsyCap) and life satisfaction [135], or via survival [132]. We define CDM as fortitude to navigate uncertainties, make difficult decisions, and embrace calculated discomfort, consistent with Rate et al.’s profile of a courageous person [136]. High CDM may enable faster pivots and capital-efficient execution under pressure—key in volatile startup environments.

*Strategic Innovativeness (STI)*: Innovativeness has been related to entrepreneurial intentions and performance (e.g., [51]), and linked to the Big Five’s Openness to Experience [57,137]. It underpins creative problem-solving, breakthrough ideas, and wealth creation [29,138], and can even catalyze competitive advantage, though it may be hindered by limited organizational support [139]. In our model, STI denotes targeted, financially aligned novelty—introducing or adjusting offerings in line with long-term strategy (cf. [140]). High STI may support capital-efficient innovation and scalable product-market fit, enhancing ROIs and exit potential.

*Transformational Leadership (TRL)*: Entrepreneurial Leadership [141] embodies innovation, proactiveness, sound decision-making, and adaptiveness [142,143]. By contrast, our TRL factor emphasizes visioning, empowering, and influence, consistent with Bass’s conception of “transformational leadership” [144] and with Yukl and Gardner’s interpersonal influence view [145]. TRL’s emphasis on articulating a compelling vision, mobilizing talent, and leading through uncertainty fits startup conditions, extends beyond conventional managerial competence [146], and is associated with startup success [147]. High TRL may support team retention, capital-efficient scaling, and execution of strategic exits.

### 4.3. Five or six factors?

Across factor retention criteria, evidence supported both five- and six-factor solutions. Parallel analysis and several CFA indices favored a five-factor structure excluding Transformational Leadership (TRL), whereas Kaiser’s eigenvalues and scree plot inspection supported a six-factor solution. Consistent with best practice, we therefore evaluated both models. The five-factor model demonstrated superior and more stable fit across all participant groups, supporting its interpretation as the more parsimonious cross-group structure of founder success characteristics. At the same time, the six-factor model showed acceptable fit, particularly within the Successful Startup Founder group, and TRL demonstrated adequate reliability and discriminant validity. We therefore retain the six-factor model as a theoretically motivated founder-specific extension, treating transformational leadership [144,146] as a contextually relevant extension rather than a uniformly expressed and fully invariant core founder characteristic across participant groups.

This distinction is theoretically meaningful. Founder leadership may emerge more strongly across venture development, shaped by venture stage, team scale, organizational complexity, repeated exposure to uncertainty, and external pressures rather than reflecting an early dispositional tendency alone [142,143]. As such, leadership may remain less differentiated or weakly expressed among aspiring entrepreneurs and managers, but salient among founders navigating growth, uncertainty, and coordination demands. The heterogeneity observed across extraction criteria and participant groups is consistent with this context-dependent interpretation of leadership in entrepreneurial settings.

Accordingly, we present five-factor results as the more parsimonious and comparatively stable cross-group structure, while retaining the six-factor structure to capture leadership-related variance associated with realized startup success. Future research should further examine the conditions under which leadership-related variance contributes incremental explanatory or predictive value across entrepreneurial contexts.

### 4.4. Comparison with prior multi-trait models

As meta-analyses confirm, associations between entrepreneurial intent and orientation scales and entrepreneurial performance outcomes are typically small (*r* < 0.30; [17,18,25,51,54,57], highlighting the limits of existing intent- and orientation-focused approaches for capturing founder-specific success characteristics. Building on this foundation, the SFSS extends prior work by incorporating items informed by practical observations and insights from experienced founders, mentors, and investors operating in high-growth startup contexts.

In contrast to Howard’s Entrepreneurial Personality Scale (EPS [20]), which integrates well-established traits associated primarily with entrepreneurial orientation, the SFSS emphasizes founder-relevant dispositions grounded in execution, opportunity realization, and sustained decision-making under uncertainty. Direct comparison with the explanatory value of Howard’s EPS is not possible, as total variance explained was not reported. The present study focuses on founders meeting predefined success benchmarks, alongside high-level corporate managers and aspiring entrepreneurs, to enable clearer group-level comparisons.

Our model also differs from Bolton and Lane’s adaptation [19] of Lumpkin and Dess’s EO framework [13] and from Staniewski’s approach [39], in that it centers on challenges specific to startup execution and founders’ direct role in navigating them. Reliance on university student samples (e.g., [19,21]) and geographically bounded samples (e.g., [39]) may partly explain differences in construct coverage and observed effect sizes across instruments. Moreover, Santos et al.’s EPAI emphasizes psychosocial readiness [21], whereas the present focus is on characteristics associated with realized business success.

This domain-specific, founder-grounded approach is intended to complement existing personality, intent, and entrepreneurial orientation measures, particularly in contexts where execution under uncertainty and resource orchestration are central to startup success.

### 4.5. Differences between successful-startup founders and corporate managers

The Regression Factor Score Analysis unveils significant differences between Successful Startup Founders (SSF) and Corporate Managers (CM) (cf. [25,33,56,57]. SSF exhibit the highest scores for all factors, indicating a strong alignment with key founders characteristics. In comparison, while CM do exhibit positive factor scores for RER and TRL, these are generally lower in magnitude compared to SSF. However, the negative factor scores for INC and VCO indicate divergence from the entrepreneurial profile as suggested by Schumpeter [29] and Pech and Cameron [126], respectively. CM associations with CDM and STI are weaker, reflecting a comparatively more risk-managed and less innovation-driven profile, consistent with prior work suggesting that corporate managers may share some entrepreneurial characteristics [18,56], but at lower intensity.

### 4.6. Differences between successful-startup founders and aspiring entrepreneurs

Comparing Successful Startup Founders (SSF) with Aspiring Entrepreneurs (AE) reveals striking disparities, diverging from Brandstätter’s view that prospective founders resemble existing successful founders [148]. AE consistently display negative factor scores across all six factors, with pronounced weak associations with RER, TRL, and STI (INC is relatively less weak). Post-hoc tests also show AE differ substantially from CM. These differences suggest that only a subset within the AE group may possess profiles comparable to those observed in successful founders or corporate managers. Also, many may explore entrepreneurship due to necessity, or non-entrepreneurial motives, while lacking key founder characteristics essential for success (cf. [149]).

### 4.7. Methodological considerations and temporal scope

The primary dataset analyzed in this study was collected between 2015 and 2017 across multiple recruitment waves. Because the present work focuses on identifying *trait-level psychological structure* rather than time-sensitive behaviors, market conditions, or short-term performance outcomes, the multi-year collection period does not undermine the validity of the scale development analyses. Trait constructs such as resilience, curiosity, opportunistic value creation, decision-making under uncertainty, innovativeness, and leadership are theorized to be relatively stable individual differences rather than transient states. Moreover, staged recruitment across independent networks and time windows likely reduced susceptibility to short-term contextual or event-driven response bias. Future research may nonetheless examine temporal stability and cohort effects through longitudinal or repeated-measures designs.

### 4.8. Practical implications

The present findings suggest that founder-related characteristics associated with realized startup success can be differentiated from entrepreneurial intent alone, with potential implications for entrepreneurial finance, founder development, and innovation policy. *(1) Improved resource allocation and investor portfolio formation –* Entrepreneurial intent, often driven by financial incentives or autonomy, does not consistently translate into realized startup success. The present results indicate that characteristics associated with realized success are unevenly distributed among individuals with entrepreneurial aspirations. The SFSS may therefore support more structured evaluation of founder-related characteristics among aspiring entrepreneurs when allocating scarce capital, mentorship, and support resources. In high-uncertainty environments characterized by high startup failure rates, such assessments may complement existing evaluation processes. *(2) More informed and potentially more equitable early-stage assessment –* Early-stage due diligence usually emphasizes idea validation, personal networks, and geographic proximity to investors, which may overlook founder-level characteristics and contribute to biased capital access. Incorporating a founder-focused framework such as the SFSS may support more structured comparisons across founders operating in different ecosystems and reduce exclusive reliance on informal network-based signals such as warm introductions or founder proximity to established investor ecosystems. *(3) Targeted training, guidance, and founder development –* SFSS profiles may also inform differentiated support strategies in educational, accelerator, or mentoring contexts. Some aspiring entrepreneurs may benefit from targeted mentoring, skills development, or network access, aligned with specific SFSS dimensions (e.g., opportunity-recognition training for lower VCO profiles or strategic planning support for lower STI profiles), while others may use such feedback for reflective career planning. Although certain characteristics may be relatively stable, others (e.g., strategic innovativeness or value-creating opportunism) are plausibly developable, suggesting possible applications in founder readiness training and development programs. *(4) Economic development and policy contexts –* At the ecosystem level, structured consideration of founder characteristics, used in conjunction with existing program criteria, may help public and private actors better understand variation in program outcomes. Structured founder-focused assessments, paired with appropriate safeguards, could inform future research on how entrepreneurship initiatives are designed and evaluated, including their potential economic impact.

The SFSS is not intended to serve as a deterministic founder-selection instrument, but rather as a structured framework that may complement existing approaches to founder evaluation, development, and entrepreneurial support.

### 4.9. Limitations and future research directions

Despite the large, diverse sample and rigorous psychometric approach, several limitations should be acknowledged. The present work does not propose that founder characteristics are sufficient determinants of startup success, but rather that they represent one important level of analysis within broader entrepreneurial, organizational, financial, and ecosystem conditions. First, *self-report and common-method bias.* The SFSS relies on self-reported data, which may introduce response biases, including social desirability or self-enhancement. Although expert and lay item review, along with a stable and interpretable factor structure, mitigate some concerns, future research should incorporate complementary data sources. Behavioral measures, experimental decision tasks, or third-party ratings (e.g., from mentors, investors, or co-founders) would enable triangulation of founder characteristics and strengthen construct validity. *Second, sampling frame and generalizability.* Although participants were drawn from over 80 countries, the sample was concentrated in the United States and Europe, and item generation relied primarily on sources from major startup hubs. Cultural, institutional, and ecosystem-stage differences may influence how founder characteristics are expressed or rewarded, potentially limiting generalizability to other contexts. Cross-cultural and cross-regional measurement invariance was not tested and remains an important direction for future validation. *Third, cross-sectional design.* The present study is observational and cross-sectional. While item selection emphasized early-emerging and relatively stable characteristics, causal inference and temporal precedence cannot be established. Longitudinal designs are required to examine whether SFSS scores prospectively relate to real-world venture outcomes (e.g., funding, growth, exit) and to assess how founder characteristics evolve across venture stages. *Fourth, instrument coverage and benchmarking.* The SFSS was not administered alongside other entrepreneurial instruments such as the Individual Entrepreneurial Orientation (IEO) or Entrepreneurial Personality Scale (EPS), limiting direct comparative evaluation. Although Big Five measures were collected to allow benchmarking against broad personality models, those analyses fall outside the scope of the present paper and will be reported separately. The study was not pre-registered; all analyses were conducted in accordance with established psychometric best practices and were conducted according to a predefined analytic plan.

Several avenues for future research follow naturally. First, longitudinal validation is needed to test whether SFSS scores predict real-world outcomes such as funding, growth, or exits in new founder cohorts. Second, multi-method approaches combining SFSS scores with behavioral tasks, peer evaluations, or investor assessments may improve predictive accuracy. Third, trainability studies should examine which SFSS dimensions can be strengthened through experience or intervention, and how such changes affect venture outcomes. Fourth, cross-cultural and sectoral validation would clarify whether the SFSS performs consistently across regions, industries, and venture types. Finally, future work should explore when transformational leadership adds predictive value, such as during scaling or crisis phases, versus contexts in which the core five-factor structure may suffice.

## 5. Conclusion

The Startup Founder Success Scale (SFSS) advances entrepreneurship research by introducing a validated, domain-specific, multi-trait measure grounded in founder narratives and expert insight and tested in a large, heterogeneous sample (N = 10,007; Successful Startup Founders n = 6,142). The six characteristics—relentless resilience, intrinsic curiosity, value-creating opportunism, courageous decision-making, strategic innovativeness, and transformational leadership—jointly account for a substantial proportion of the common variance captured by the scale and reliably distinguish successful founders from corporate managers and aspiring entrepreneurs.

By clarifying founder characteristics associated with realized startup success beyond general managerial competence or entrepreneurial intent, the SFSS helps explain limitations of broad personality models in startup contexts. Practically, it provides a domain-specific framework for assessing founder-related characteristics that may inform entrepreneurial research, education, targeted training programs, founder evaluation, and capital allocation processes across public and private contexts.

Theoretically, the SFSS situates founder characteristics within a conceptual model linking decision-making under uncertainty and resource orchestration to venture execution and growth. By aligning measurement more closely with real-world founder contexts, this work helps narrow the gap between entrepreneurship theory and practice and provides a foundation for future research examining how founder characteristics relate to entrepreneurial outcomes in innovation-driven ventures.

## Supporting information

S1 Fig Raw Pearson correlation matrix for the retained 31 items. Each cell represents the Pearson correlation coefficient between pairs of retained items. Darker shades of green indicate stronger positive correlations, whereas darker shades of red indicate comparatively weaker correlations.
(TIF)

S2 Fig Q–Q plots across the six founder characteristics for the three participant groups. Q–Q plots are shown for Successful Startup Founders (SSF), Corporate Managers (CM), and Aspiring Entrepreneurs (AE) across the six SFSS dimensions to visually assess deviations from normality. The diagonal reference line indicates the expected distribution under normality.
(TIF)

S3 Fig Mean factor scores across participant groups. Mean scores for the six SFSS traits are shown for Successful Startup Founders (SSF), Corporate Managers (CM), and Aspiring Entrepreneurs (AE). Although items were rated on a 1–5 Likert scale, the displayed x-axis focuses on the 2.5–4.5 interval, where the observed scores were concentrated, to improve visualization of between-group differences. Both statistically significant and non-significant group differences are displayed for comparative purposes.
(TIF)

S1 Table Expert panel ratings of item-level (CVI-I) and scale-level (CVI-S) content relevance, appropriateness, and clarity. Items with two or more values below the suggested cut-off thresholds (underlined) were eliminated, whereas items with italicized values required minor revisions.
(DOCX)

S2 Table Exploratory factor analysis (EFA) for the Successful Startup Founders group. Factor loadings exceeding.50 are shown in bold, and the identified cross-loading is underlined.
(DOCX)

S3 Table Kolmogorov-Smirnov tests of normality across factors and participant groups. Results indicated non-normal distribution across all factors and groups.
(DOCX)

S4 Table Skewness and kurtosis statistics across factors and participant groups. (DOCX)

S5 Table Tamhane’s T2 and Games-Howell post-hoc tests for multiple comparisons across participant groups. The asterisk (*) denotes statistical significance at the 0.05 level. Tests were conducted on regression factor scores derived from the EFA solution.
(DOCX)
