# Supplementary figures and images for: Development and validation of a domain-specific scale of founder characteristics associated with startup success
Source: PLoS One. 2026 Jun 26;21(6):e0351970. doi: 10.1371/journal.pone.0351970 (PMC13308860; doi:10.1371/journal.pone.0351970)

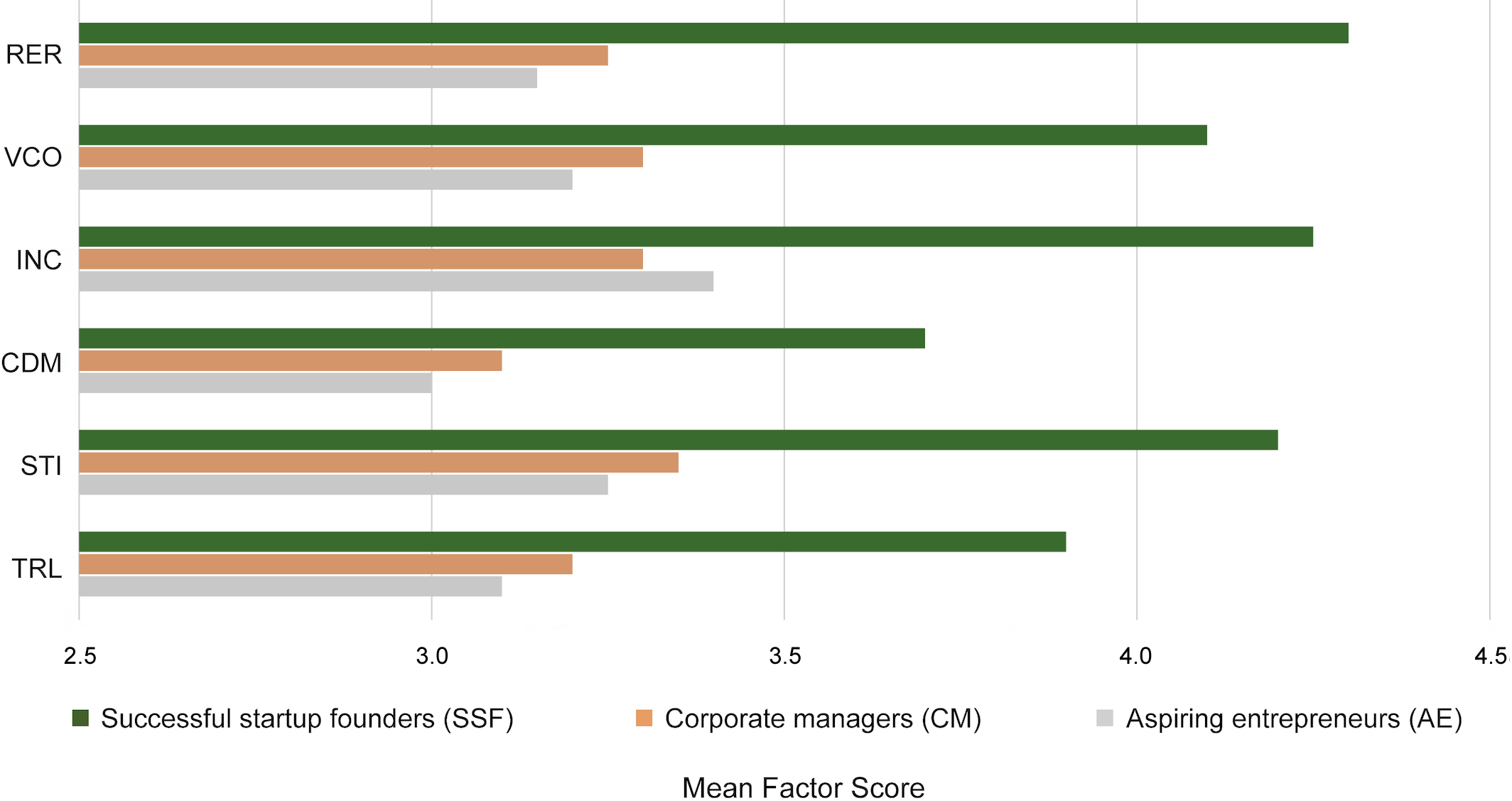

Supplement: S3 Fig — Mean scores for the six SFSS traits are shown for Successful Startup Founders (SSF), Corporate Managers (CM), and Aspiring Entrepreneurs (AE). Although items were rated on a 1–5 Likert scale, the displayed x-axis focuses on the 2.5–4.5 interval, where the observed scores were concentrated, to improve visualization of between-group differences. Both statistically significant and non-significant group differences are displayed for comparative purposes. (TIF) [file pone.0351970.s003.tif]
