# Supplementary material for: Development and validation of a domain-specific scale of founder characteristics associated with startup success
Source: PLoS One. 2026 Jun 26;21(6):e0351970. doi: 10.1371/journal.pone.0351970 (PMC13308860; doi:10.1371/journal.pone.0351970)
Supplement: S1 Table — Items with two or more values below the suggested cut-off thresholds (underlined) were eliminated, whereas items with italicized values required minor revisions. (DOCX) [file pone.0351970.s004.docx]

**S1 Table. Expert panel ratings of item-level (CVI-I) and scale-level (CVI-S) content relevance, appropriateness, and clarity.**

| **Dimension** | **Item** | **Domain Experts Panel (CVI-I)** | | | **Lay Experts Panel (CVI-I)** | | |
| --- | --- | --- | --- | --- | --- | --- | --- |
|  |  | **Relevant** | **Appropriate** | **Clear** | **Relevant** | **Appropriate** | **Clear** |
| Curiosity | 1 | 1.00 | 1.00 | 1.00 | 1.00 | 1.00 | 1.00 |
|  | 2 | 1.00 | 1.00 | 1.00 | 1.00 | 1.00 | 1.00 |
|  | 3 | 1.00 | 1.00 | 1.00 | 1.00 | 1.00 | 1.00 |
|  | 4 | .90 | .90 | .90 | .90 | *.80* | *.80* |
|  | 5 | 1.00 | 1.00 | 1.00 | .90 | .90 | *.80* |
|  | 6 | 1.00 | 1.00 | 1.00 | 1.00 | .90 | .90 |
|  | 7 | 1.00 | .90 | *.80* | .90 | .90 | .90 |
|  | CVI-D/Ave | .99 | .97 | .96 | .96 | .93 | .91 |
| Innovative ness | 1 | 1.00 | 1.00 | 1.00 | 1.00 | 1.00 | 1.00 |
|  | 2 | 1.00 | 1.00 | 1.00 | 1.00 | 1.00 | 1.00 |
|  | 3 | 1.00 | 1.00 | 1.00 | 1.00 | 1.00 | 1.00 |
|  | 4 | .90 | .90 | .90 | 1.00 | 1.00 | 1.00 |
|  | 5 | 1.00 | 1.00 | 1.00 | .90 | 1.00 | .90 |
|  | 6 | 1.00 | 1.00 | 1.00 | .90 | .90 | .90 |
|  | 7 | .90 | .90 | .90 | .90 | .90 | .90 |
|  | CVI-D/Ave | .97 | .97 | .97 | .96 | .97 | .96 |
| Resilience | 1 | 1.00 | 1.00 | 1.00 | 1.00 | 1.00 | 1.00 |
|  | 2 | 1.00 | 1.00 | 1.00 | 1.00 | 1.00 | 1.00 |
|  | 3 | 1.00 | 1.00 | 1.00 | .90 | .90 | .90 |
|  | 4 | 1.00 | 1.00 | 1.00 | 1.00 | 1.00 | 1.00 |
|  | 5 | .90 | .90 | .90 | .90 | .90 | .90 |
|  | 6 | 1.00 | 1.00 | 1.00 | 1.00 | 1.00 | 1.00 |
|  | 7 | 1.00 | 1.00 | 1.00 | 1.00 | 1.00 | 1.00 |
|  | CVI-D/Ave | .99 | .99 | .99 | .97 | .97 | .97 |
| Emotional Stability | 1 | .90 | .90 | .90 | *.80* | *.80* | *.80* |
|  | 2 | .90 | .90 | .90 | .90 | .90 | .90 |
|  | 3 | *.80* | .70 | *.80* | .70 | *.80* | .90 |
|  | 4 | .90 | .90 | .90 | 1.00 | 1.00 | 1.00 |
|  | 5 | .90 | .90 | .90 | *.80* | *.80* | *.80* |
|  | 6 | .70 | *.80* | .90 | .70 | *.80* | *.80* |
|  | 7 | .90 | .90 | .90 | 1.00 | .90 | .90 |
|  | CVI-D/Ave | *.86* | *.86* | *.89* | *.84* | *.86* | *.87* |
| Leadership | 1 | 1.00 | 1.00 | 1.00 | .90 | .90 | .90 |
|  | 2 | .70 | *.80* | *.80* | .70 | .70 | *.80* |
|  | 3 | 1.00 | 1.00 | *.80* | .90 | .90 | .90 |
|  | 4 | 1.00 | 1.00 | 1.00 | 1.00 | 1.00 | 1.00 |
|  | 5 | 1.00 | 1.00 | *.80* | .90 | .90 | .90 |
|  | 6 | 1.00 | 1.00 | 1.00 | 1.00 | 1.00 | 1.00 |
|  | 7 | 1.00 | 1.00 | 1.00 | 1.00 | 1.00 | 1.00 |
|  | CVI-D/Ave | 1.00 | .97 | .96 | .91 | .91 | .93 |
| Value Creation / Opportunism | 1 | 1.00 | 1.00 | 1.00 | 1.00 | 1.00 | 1.00 |
|  | 2 | 1.00 | .90 | .90 | .90 | *.80* | *.80* |
|  | 3 | 1.00 | 1.00 | 1.00 | 1.00 | 1.00 | .90 |
|  | 4 | 1.00 | .90 | *.80* | 1.00 | 1.00 | .90 |
|  | 5 | 1.00 | 1.00 | 1.00 | .90 | .90 | .90 |
|  | 6 | 1.00 | .90 | .70 | 1.00 | .70 | .90 |
|  | 7 | 1.00 | 1.00 | 1.00 | 1.00 | 1.00 | 1.00 |
|  | CVI-D/Ave | 1.00 | .96 | .91 | .97 | .91 | .91 |
| Courage | 1 | 1.00 | 1.00 | 1.00 | 1.00 | 1.00 | 1.00 |
|  | 2 | 1.00 | 1.00 | 1.00 | 1.00 | 1.00 | 1.00 |
|  | 3 | 1.00 | *.80* | .70 | *.80* | *.80* | *.80* |
|  | 4 | 1.00 | 1.00 | 1.00 | 1.00 | 1.00 | 1.00 |
|  | 5 | 1.00 | 1.00 | 1.00 | 1.00 | 1.00 | 1.00 |
|  | 6 | 1.00 | 1.00 | 1.00 | 1.00 | 1.00 | 1.00 |
|  | 7 | 1.00 | 1.00 | 1.00 | 1.00 | 1.00 | .90 |
|  | CVI-D/Ave | 1.00 | .97 | .96 | .97 | .97 | .96 |
| CVI-S/Ave | | .97 | .96 | .94 | .94 | .93 | .93 |

Items with two or more values below the suggested cut-off thresholds (underlined) were eliminated, whereas items with italicized values required minor revisions.
