# Supplementary material for: Development and validation of a domain-specific scale of founder characteristics associated with startup success
Source: PLoS One. 2026 Jun 26;21(6):e0351970. doi: 10.1371/journal.pone.0351970 (PMC13308860; doi:10.1371/journal.pone.0351970)
Supplement: S2 Table — Factor loadings exceeding.50 are shown in bold, and the identified cross-loading is underlined. (DOCX) [file pone.0351970.s005.docx]

**S2 Table. Exploratory factor analysis (EFA) for the Successful Startup Founders group.**

| **Items** | **Factors** | | | | | |
| --- | --- | --- | --- | --- | --- | --- |
|  | **1** | **2** | **3** | **4** | **5** | **6** |
| (RER) Item 15 | **.916** | -.057 | -.024 | -.026 | .012 | -.010 |
| (RER) Item 16 | **.914** | .011 | -.063 | -.053 | .054 | -.012 |
| (RER) Item 18 | **.768** | .086 | .101 | -.003 | .000 | -.060 |
| (RER) Item 17 | **.767** | .097 | -.067 | -.067 | .059 | .011 |
| (RER) Item 19 | **.746** | -.071 | .042 | .003 | .004 | .105 |
| (RER) Item 20 | **.627** | .170 | -.001 | .184 | -.027 | -.026 |
| (VCO) Item 38 | -.044 | **.884** | -.101 | -.060 | .039 | .157 |
| (VCO) Item 36 | -.021 | **.826** | .207 | .026 | -.064 | -.074 |
| (VCO) Item 40 | .189 | **.757** | .008 | .100 | -.098 | -.075 |
| (VCO) Item 39 | -.192 | **.645** | -.093 | -.004 | .177 | .274 |
| (VCO) Item 41 | .198 | **.622** | .021 | -.003 | .032 | -.036 |
| (INC) Item 22 | -.007 | -.037 | **.868** | -.070 | .056 | -.042 |
| (INC) Item 23 | -.007 | -.008 | **.802** | .084 | -.078 | -.035 |
| (INC) Item 24 | -.036 | .041 | **.779** | -.024 | .090 | -.015 |
| (INC) Item 26 | .018 | -.012 | **.743** | .001 | .012 | .092 |
| (INC) Item 25 | .031 | .204 | **.578** | -.060 | .117 | .126 |
| (CDM) Item 2 | -.078 | .062 | .054 | **.860** | -.079 | -.080 |
| (CDM) Item 1 | .031 | .116 | .017 | **.752** | -.166 | .003 |
| (CDM) Item 6 | .017 | -.018 | .022 | **.746** | .004 | .024 |
| (CDM) Item 4 | -.027 | -.030 | -.005 | **.663** | .168 | .115 |
| (CDM) Item 5 | -.012 | -.087 | -.125 | **.655** | .136 | -.042 |
| (STI) Item 33 | -.030 | -.017 | .108 | -.020 | **.859** | -.111 |
| (STI) Item 31 | -.083 | .004 | .076 | -.031 | **.848** | -.004 |
| (STI) Item 30 | .142 | .228 | -.102 | -.033 | **.759** | -.188 |
| (STI) Item 32 | .172 | -.186 | -.055 | .127 | **.532** | .189 |
| (STI) Item 35 | .120 | -.200 | .112 | .154 | **.519** | .185 |
| (TRL) Item 8 | -.053 | .120 | .028 | .036 | -.142 | **.768** |
| (TRL) Item 9 | .188 | -.144 | .026 | -.010 | -.096 | **.751** |
| (TRL) Item 10 | -.114 | .218 | -.038 | -.011 | .071 | **.660** |
| (TRL) Item 12 | .374 | -.066 | .044 | -.075 | -.173 | **.612** |
| (TRL) Item 11 | -.013 | .165 | -.049 | -.019 | .147 | **.554** |

Factor loadings exceeding .50 are shown in bold, and the identified cross-loading is underlined.
