# Supplementary material for: Development and validation of a domain-specific scale of founder characteristics associated with startup success
Source: PLoS One. 2026 Jun 26;21(6):e0351970. doi: 10.1371/journal.pone.0351970 (PMC13308860; doi:10.1371/journal.pone.0351970)
Supplement: S3 Table — Results indicated non-normal distribution across all factors and groups. (DOCX) [file pone.0351970.s006.docx]

**S3 Table. Kolmogorov-Smirnov tests of normality across factors and participant groups.**

| **Factors** | **Group** | **Kolmogorov-Smirnov** | | |
| --- | --- | --- | --- | --- |
|  |  | **Statistic** | **df** | **Sig.** |
| Relentless Resilience (RER) | 1 (SSF) | 0.11 | 6142 | <.001 |
|  | 2 (CM) | 0.12 | 2004 | <.001 |
|  | 3 (AE) | 0.12 | 1861 | <.001 |
| Value-Creating Opportunism (VCO) | 1 (SSF) | 0.09 | 6142 | <.001 |
|  | 2 (CM) | 0.11 | 2004 | <.001 |
|  | 3 (AE) | 0.11 | 1861 | <.001 |
| Intrinsic Curiosity (INC) | 1 (SSF) | 0.12 | 6142 | <.001 |
|  | 2 (CM) | 0.20 | 2004 | <.001 |
|  | 3 (AE) | 0.12 | 1861 | <.001 |
| Courageous Decision-Making (CDM) | 1 (SSF) | 0.08 | 6142 | <.001 |
|  | 2 (CM) | 0.11 | 2004 | <.001 |
|  | 3 (AE) | 0.09 | 1861 | <.001 |
| Strategic Innovativeness (STI) | 1 (SSF) | 0.09 | 6142 | <.001 |
|  | 2 (CM) | 0.14 | 2004 | <.001 |
|  | 3 (AE) | 0.12 | 1861 | <.001 |
| Transformational Leadership (TRL) | 1 (SSF) | 0.11 | 6142 | <.001 |
|  | 2 (CM) | 0.10 | 2004 | <.001 |
|  | 3 (AE) | 0.08 | 1861 | <.001 |

Results indicated non-normal distribution across all factors and groups.
