# Supplementary material for: Development and validation of a domain-specific scale of founder characteristics associated with startup success
Source: PLoS One. 2026 Jun 26;21(6):e0351970. doi: 10.1371/journal.pone.0351970 (PMC13308860; doi:10.1371/journal.pone.0351970)
Supplement: S4 Table — (DOCX) [file pone.0351970.s007.docx]

**S4 Table. Skew and kurtosis statistics across factors and participant groups.**

| **Factors** | **Skew** | | | **Kurtosis** | | |
| --- | --- | --- | --- | --- | --- | --- |
|  | **Group 1 (SSF)** | **Group 2 (CM)** | **Group 3 (AE)** | **Group 1 (SSF)** | **Group 2 (CM)** | **Group 3 (AE)** |
| Relentless Resilience (RER) | -0.74 | -0.69 | -0.99 | 0.51 | 0.45 | 2.17 |
| Value-Creating Opportunism (VCO) | -0.52 | -0.77 | -0.39 | 1.02 | 2.45 | 0.45 |
| Intrinsic Curiosity (INC) | -0.43 | -0.15 | -0.23 | 0.45 | 0.10 | -0.46 |
| Courageous Decision-Making (CDM) | -0.29 | -0.58 | -0.40 | 0.29 | 0.12 | 0.06 |
| Strategic Innovativeness (STI) | -0.56 | -0.19 | -0.28 | 0.45 | 0.06 | 0.01 |
| Transformational Leadership (TRL) | -0.50 | -0.39 | -0.17 | 0.07 | 0.53 | -0.32 |
