# Supplementary material for: Development and validation of a domain-specific scale of founder characteristics associated with startup success
Source: PLoS One. 2026 Jun 26;21(6):e0351970. doi: 10.1371/journal.pone.0351970 (PMC13308860; doi:10.1371/journal.pone.0351970)
Supplement: S5 Table — The asterisk (*) denotes statistical significance at the 0.05 level. Tests were conducted on regression factor scores derived from the EFA solution. (DOCX) [file pone.0351970.s008.docx]

**S5 Table. Tamhane**’**s T2 and Games-Howell post-hoc tests for multiple comparisons across participant groups.**

| **Dependent Variable** | **Post-Hoc Test** | **Group** | **Group** | **Mean Difference (I-J)** | **Std. Error** | **Sig.** |
| --- | --- | --- | --- | --- | --- | --- |
| **Relentless Resilience (RER)** | Tamhane | 1 (SSF) | 2 (CM) | 0.009 | 0.013 | 0.882 |
|  |  |  | 3 (AE) | .107* | 0.015 | <.001 |
|  |  | 2 (CM) | 1 (SSF) | -0.009 | 0.013 | 0.882 |
|  |  |  | 3 (AE) | .099* | 0.018 | <.001 |
|  |  | 3 (AE) | 1 (SSF) | -.107* | 0.015 | <.001 |
|  |  |  | 2 (CM) | -.099* | 0.018 | <.001 |
|  | Games-Howell | 1 (SSF) | 2 (CM) | 0.009 | 0.013 | 0.787 |
|  |  |  | 3 (AE) | .107* | 0.015 | <.001 |
|  |  | 2 (CM) | 1 (SSF) | -0.009 | 0.013 | 0.787 |
|  |  |  | 3 (AE) | .099* | 0.018 | <.001 |
|  |  | 3 (AE) | 1 (SSF) | -.107* | 0.015 | <.001 |
|  |  |  | 2 (CM) | -.099* | 0.018 | <.001 |
| **Value-Creating Opportunism (VCO)** | Tamhane | 1 (SSF) | 2 (CM) | .091* | 0.013 | <.001 |
|  |  |  | 3 (AE) | .091* | 0.014 | <.001 |
|  |  | 2 (CM) | 1 (SSF) | -.091* | 0.013 | <.001 |
|  |  |  | 3 (AE) | -0.00 | 0.017 | 1 |
|  |  | 3 (AE) | 1 (SSF) | -.091* | 0.014 | <.001 |
|  |  |  | 2 (CM) | 0.00 | 0.017 | 1 |
|  | Games-Howell | 1 (SSF) | 2 (CM) | .091* | 0.013 | <.001 |
|  |  |  | 3 (AE) | .091* | 0.014 | <.001 |
|  |  | 2 (CM) | 1 (SSF) | -.091* | 0.013 | <.001 |
|  |  |  | 3 (AE) | -0.000 | 0.017 | 1 |
|  |  | 3 (AE) | 1 (SSF) | -.091* | 0.014 | <.001 |
|  |  |  | 2 (CM) | 0.000 | 0.017 | 1 |
| **Intrinsic Curiosity (INC)** | Tamhane | 1 (SSF) | 2 (CM) | .108* | 0.013 | <.001 |
|  |  |  | 3 (AE) | .080* | 0.013 | <.001 |
|  |  | 2 (CM) | 1 (SSF) | -.108* | 0.013 | <.001 |
|  |  |  | 3 (AE) | -0.028 | 0.016 | 0.232 |
|  |  | 3 (AE) | 1 (SSF) | -.081* | 0.013 | <.001 |
|  |  |  | 2 (CM) | 0.028 | 0.016 | 0.232 |
|  | Games-Howell | 1 (SSF) | 2 (CM) | .108* | 0.017 | <.001 |
|  |  |  | 3 (AE) | .081* | 0.013 | <.001 |
|  |  | 2 (CM) | 1 (SSF) | -.108* | 0.0127 | <.001 |
|  |  |  | 3 (AE) | -0.028 | 0.016 | 0.195 |
|  |  | 3 (AE) | 1 (SSF) | -.081* | 0.013 | <.001 |
|  |  |  | 2 (CM) | 0.028 | 0.016 | 0.195 |
| **Courageous Decision-Making (CDM)** | Tamhane | 1 (SSF) | 2 (CM) | .052* | 0.017 | 0.005 |
|  |  |  | 3 (AE) | .148* | 0.019 | <.001 |
|  |  | 2 (CM) | 1 (SSF) | -.052* | 0.017 | 0.005 |
|  |  |  | 3 (AE) | .096* | 0.023 | <.001 |
|  |  | 3 (AE) | 1 (SSF) | -.148* | 0.019 | <.001 |
|  |  |  | 2 (CM) | -.096* | 0.023 | <.001 |
|  | Games-Howell | 1 (SSF) | 2 (CM) | .052* | 0.017 | 0.005 |
|  |  |  | 3 (AE) | .148* | 0.019 | <.001 |
|  |  | 2 (CM) | 1 (SSF) | -.052* | 0.017 | 0.005 |
|  |  |  | 3 (AE) | .096* | 0.023 | <.001 |
|  |  | 3 (AE) | 1 (SSF) | -.148* | 0.019 | <.001 |
|  |  |  | 2 (CM) | -.096* | 0.023 | <.001 |
| **Strategic Innovativeness (STI)** | Tamhane | 1 (SSF) | 2 (CM) | .073* | 0.013 | <.001 |
|  |  |  | 3 (AE) | .229* | 0.015 | <.001 |
|  |  | 2 (CM) | 1 (SSF) | -.073* | 0.013 | <.001 |
|  |  |  | 3 (AE) | .156* | 0.017 | <.001 |
|  |  | 3 (AE) | 1 (SSF) | -.229* | 0.015 | <.001 |
|  |  |  | 2 (CM) | -.156* | 0.017 | <.001 |
|  | Games-Howell | 1 (SSF) | 2 (CM) | .073* | 0.013 | <.001 |
|  |  |  | 3 (AE) | .229* | 0.015 | <.001 |
|  |  | 2 (CM) | 1 (SSF) | -.073* | 0.013 | <.001 |
|  |  |  | 3 (AE) | .156* | 0.017 | <.001 |
|  |  | 3 (AE) | 1 (SSF) | -.229* | 0.015 | <.001 |
|  |  |  | 2 (CM) | -.156* | 0.017 | <.001 |
| **Transformational Leadership (TRL)** | Tamhane | 1 (SSF) | 2 (CM) | .040* | 0.016 | 0.044 |
|  |  |  | 3 (AE) | .252* | 0.019 | <.001 |
|  |  | 2 (CM) | 1 (SSF) | -.040* | 0.016 | 0.044 |
|  |  |  | 3 (AE) | .212* | 0.022 | <.001 |
|  |  | 3 (AE) | 1 (SSF) | -.252* | 0.019 | <.001 |
|  |  |  | 2 (CM) | -.212* | 0.022 | <.001 |
|  | Games-Howell | 1 (SSF) | 2 (CM) | .040* | 0.016 | 0.039 |
|  |  |  | 3 (AE) | .252* | 0.019 | <.001 |
|  |  | 2 (CM) | 1 (SSF) | -.040* | 0.016 | 0.039 |
|  |  |  | 3 (AE) | .212* | 0.022 | <.001 |
|  |  | 3 (AE) | 1 (SSF) | -.252* | 0.019 | <.001 |
|  |  |  | 2 (CM) | -.212* | 0.022 | <.001 |

The asterisk (*) denotes statistical significance at the 0.05 level.
