# Supplementary material for: Development and validation of a domain-specific scale of founder characteristics associated with startup success
Source: PLoS One. 2026 Jun 26;21(6):e0351970. doi: 10.1371/journal.pone.0351970 (PMC13308860; doi:10.1371/journal.pone.0351970)
Supplement: Attachment — Submitted filename: Minor Comments.docx [file pone.0351970.s009.docx]

**Minor Comments:**

1.	Abstract: Well written

2.	Manuscript is generally well written but:

A. Some sections are overly long (especially introduction)

B. Repetition in discussion

Scope for reducing the word count

3.	Tables and figures should be simplified.

4. Clarify:

A. Difference between 5-factor vs 6-factor model

B. Role of leadership more explicitly

5.	Reference: Ok
