# Supplementary material for: Development and validation of a domain-specific scale of founder characteristics associated with startup success
Source: PLoS One. 2026 Jun 26;21(6):e0351970. doi: 10.1371/journal.pone.0351970 (PMC13308860; doi:10.1371/journal.pone.0351970)
Supplement: Attachment — Submitted filename: SFSS_PlosOne_LetterToReviewers_28May2026_ff.docx [file pone.0351970.s011.docx]

We thank the Reviewers and the Managing Editor for their careful and constructive evaluations of the manuscript. Below, we address both the reviewer comments and the additional editorial recommendations, and believe the revisions have improved the manuscript’s clarity, transparency, and interpretability while preserving the core structure and contribution of the SFSS framework.

In response to the reviews and editorial comments, we have: (i) clarified the conceptual distinction between the five- and six-factor solutions and aligned related claims throughout the manuscript; (ii) conducted additional measurement invariance analyses across Successful Startup Founders, Corporate Managers, and Aspiring Entrepreneurs; (iii) refined wording throughout the manuscript to better distinguish discriminative validity from predictive validity and to avoid overstating implications of the cross-sectional design; (iv) clarified and expanded selected methodological, interpretative, and practical implication sections; and (v) updated the Data Availability statement to clarify procedures for accessing de-identified data.

We also describe below how we addressed the Journal Requirements and additional editorial recommendations. Journal Requirements, reviewer comments, and editorial comments are reproduced below in italics, followed by our responses in regular text. All manuscript changes are shown using track changes.

**Response to Journal Requirements**

*1. PLOS ONE formatting requirements*

Response to Point 1: The title page, manuscript, figures, supporting information files, file naming conventions, and section titles were reviewed and updated to comply with PLOS ONE formatting requirements and submission guidelines.

*2. Data availability statement and institutional contact point*

Response to Point 2: Response to Point 2: The Data Availability Statement was revised to replace the previous author-based contact with a non-author institutional contact point, in accordance with PLOS ONE Data Policy requirements. The revised statement now includes the OSF repository citation, the institutional contact information for data access requests, and details regarding access to the de-identified dataset under institutional oversight.

*3. Ethics statement placement*

Response to Point 3: The ethics statement was reviewed and retained only within the Methods section of the manuscript, in accordance with journal requirements.

*4. Figure quality*

Response to Point 4: New high-resolution versions of Figures 1–3 and Supporting Figures 1–3 were prepared and uploaded in accordance with PLOS ONE figure requirements.

*5. Supporting Information file types and legends*

Response to Point 5: Supporting figures were re-uploaded using the correct “Supporting Information” file designation. Corresponding legends for all Supporting Information files were added to the manuscript after the reference list.

*6. Reviewer-suggested references*

Response to Point 6: The reviewer-suggested publication was reviewed, found relevant to the present study, and incorporated into the revised manuscript.

*7. Reference list review*

Response to Point 7: The reference list was reviewed for completeness, accuracy, and compliance with PLOS ONE formatting requirements. No retracted references are included in the revised manuscript. In addition, in-text citations and the reference list were revised to conform to PLOS ONE’s Vancouver citation style, including conversion of author–date citations to numbered citations where applicable.

**Additional Editor Comments:**
 *I congratulate you on the work you have done so far, however, your research needs more work. I suggest you read the reviewers' contributions and act accordingly. In addition to the fact that in my opinion the study will improve with more work in the Implications section.*

Response to Editor Comment:

We revised Section 4.8 (“Practical implications”, pp.44-45) to strengthen the connection between the study findings and their potential implications for entrepreneurial finance, founder development, and innovation policy. The revised section now more clearly distinguishes founder characteristics associated with realized startup outcomes from entrepreneurial intent alone, clarifies potential applications of the SFSS framework in evaluation, mentoring, training, and ecosystem-support contexts, and includes more concrete examples linked to specific SFSS dimensions (e.g., opportunity-recognition training or strategic-planning support). We also refined the wording throughout the section to better define the intended scope and boundaries of interpretation and application.

**Response to Reviewers Comments**

**Reviewer #1**

*Overall, this is a promising and potentially high-impact scale development paper. I recommend focusing revisions on:*

*(i) clarifying the factor solution and aligning claims.*

*(ii) addressing measurement invariance (or appropriately tempering group comparison interpretations), and*

*(iii) improving transparency for reproducibility and practical use.*

Response to Comment 1:

We revised Section 4.3 (“Five or six factors?”, pp. 40–41) to clarify the conceptual and statistical distinction between the five- and six-factor solutions. The revised text now describes the five-factor structure as the more parsimonious cross-group representation of founder success characteristics, while positioning the six-factor structure as a theoretically motivated founder-specific extension that captures transformational leadership within entrepreneurial contexts. Within the same section, we also expanded the interpretation of transformational leadership to clarify its potentially context-dependent and developmentally expressed nature across venture stages, organizational complexity, and entrepreneurial contexts.

In addition, several passages throughout the manuscript were revised to better align interpretative claims with the underlying cross-sectional design. In particular, wording related to “founder selection” and “early identification of startup founders with high success potential” was revised toward more cautious phrasing focused on the structured evaluation and differentiation of founder-related characteristics.

Response to Comment 2:
We conducted additional multi-group measurement invariance analyses across Successful Startup Founders (SSF), Corporate Managers (CM), and Aspiring Entrepreneurs (AE) using the final six-factor, 31-item model. Configural, metric, scalar, and strict invariance models were evaluated. The results supported substantial cross-group stability of the SFSS factor structure, with only modest changes in practical fit indices across increasingly constrained models. Although some deterioration in fit was observed at the scalar and strict levels, the overall pattern supported general comparability of the latent structure while also indicating some group-specific variation in item intercepts and residual variances.

A new subsection titled “3.1.3.1 Measurement Invariance Across Entrepreneurial and Comparison Groups” and a corresponding table reporting invariance fit indices were added to the Results section (pp. 28–29). We also added a brief methodological description of the invariance procedure in Section 2.5.3 (“Confirmatory factor analysis”, p. 22). Given the large sample size (N = 10,007), practical fit indices were prioritised when evaluating invariance models due to the known sensitivity of chi-square statistics under large-N conditions. Corresponding methodological references on measurement invariance were also added to the References list.

In addition, earlier statements indicating that measurement invariance analyses were deferred to future validation studies were removed or revised in Sections 2.1 and 2.2 to reflect the inclusion of the present invariance analyses.

Response to Comment 3:
We revised several sections of the manuscript to improve clarity, interpretability, and practical usability of the SFSS framework and development process. Section 2.2 (“Dimensions identification and initial item generation”) was expanded to more clearly describe how founder autobiographies, practitioner interviews, investor perspectives, and related founder materials informed dimensional development and item generation (p. 16). Section 3.2 (“Interpreting the extracted factors”) was also revised to include representative example item wording for each factor to improve interpretability of the extracted dimensions (pp. 30–31).

In addition, as noted above in our response to Comment 1, we further clarified the distinction between the five- and six-factor solutions in Section 4.3 and revised several passages throughout the manuscript to more clearly distinguish discriminative validity from predictive validity. We also made several editorial and reporting updates throughout the manuscript, including revisions to the Data Availability statement in accordance with journal requirements (see also our response to the Journal Requirements).

**Reviewer #2:**

*This manuscript is well structured. It is methodologically sound and focus on domain specific scale that measure founder characteristics that are further linked to startup success. The topic is mainly relevant to entrepreneurship research and contribute to organizational research. The study has meaningful academic value. The sample size is very large so the systematic scale development was done. Expert review was use. Exploratory and confirmatory factor analysis, reliability testing is used. The methods strengthen the credibility of study. Overall the study is strong and suitable for publication after minor revisions.*

*1. However, some claims about predictive utility are strong but these claims are to be interpreted cautiously.*

*2. For future longitudinal data can be used for validation.*

*3. Data availability is data can be shared through the public repositories; the anonymised datasets are recommended for sharing.*

Response to Comment 1:

Several passages throughout the manuscript were revised to more clearly distinguish discriminative validity from predictive validity and to avoid overstating implications of the present cross-sectional design. Specifically, in the Abstract (p. 2) we changed “Early identification of startup founders with high success potential” to “Early differentiation of startup founders with high success potential” and revised references to “founder selection” toward more cautious wording focused on the structured evaluation of founder-related characteristics.

In the Introduction (pp. 3 and 5), several predictive and selection-oriented statements were softened, including replacing deterministic phrasing such as “can materially improve” with more cautious wording (“may improve”), revising references to “identifying high-potential startup founders,” and replacing the statement that limitations “can leave high-potential founders under-identified and resources misallocated” with wording indicating that such limitations “may contribute to founder profiles associated with strong venture outcomes being overlooked and resources being allocated inefficiently.”

Several conceptual statements in the Discussion were revised to avoid deterministic interpretations of founder characteristics and entrepreneurial outcomes (e.g., replacing “founder characteristics shape” with “founder characteristics are likely to influence”, p. 37).

In addition, the practical framing in the Conclusion was also revised by replacing wording referring to an “objective, early-stage assessment framework” supporting “founder selection” with more cautious language describing the SFSS as a “domain-specific framework for assessing founder-related characteristics” that may help inform founder evaluation and capital-allocation processes (pp. 47-48).

Response to Comment 2:

We agree that future longitudinal analyses will be important for further validation of the SFSS framework. The need for longitudinal validation was already acknowledged in the original manuscript, including in the Abstract, Introduction, Methodological Considerations, and Limitations/Future Directions sections. In the revised manuscript, several passages were further refined to more clearly distinguish discriminative validity from predictive validity and to avoid interpretations extending beyond the present cross-sectional design (see also our response to Comment 1 above).

For example, the revised Introduction now states that the SFSS provides “a foundation for future longitudinal studies” while emphasizing that the present work focuses on founder profiles associated with realized startup success rather than prospective prediction (p. 11).

Response to Comment 3:

We refer the reviewer to our response to the Journal Requirements (Point 2), where we describe revisions to the Data Availability statement addressing this issue.

**Reviewer #3**

*Reviewer #3: Minor Comments:
1. Abstract: Well written
2. Manuscript is generally well written but:
A. Some sections are overly long (especially introduction)
B. Repetition in discussion
Scope for reducing the word count
3. Tables and figures should be simplified.
4. Clarify:
A. Difference between 5-factor vs 6-factor model
B. Role of leadership more explicitly
5. Reference: Ok*

Response to Comment 2A and 2B:
We have carefully revised the Introduction and Discussion sections to further improve conciseness, reduce repetition, and streamline several extended conceptual and methodological passages without changing any core conceptual arguments or interpretation of the findings.

Specifically, in the Introduction (pp. 3-11), we have shortened and consolidated discussions of prior entrepreneurial personality and orientation instruments, reduced repetitive comparisons between entrepreneurial intention and realized startup success, simplified operational definition sections, and condensed examples of heterogeneous entrepreneurial samples used in prior research.

In addition, in the Discussion (pp. 36-47), we have reduced repetition across sections discussing broad personality models, entrepreneurial intention frameworks, and founder-specific characteristics. We have also streamlined several interpretive passages, reduced repetitive effect-size interpretations, and shortened repeated practical implications related to scaling, founder evaluation, and resource allocation.

These revisions now help to reduce overall manuscript length while preserving the conceptual and theoretical contributions of the study.

Response to Comment 3:
We reviewed all tables and figures for readability, presentation clarity, and potential simplification. Overall, the tables and figures were already relatively streamlined, and no major structural simplifications or analytical changes were introduced, as further reduction could risk loss of relevant methodological or interpretative information.

Nevertheless, several presentation refinements were implemented to improve clarity and readability. Figure 1 (scree plot) was revised to improve interpretability of the factor-retention criteria and corresponding visual reference markers. In addition, axis labels, legends, captions, and formatting details were added or refined across figures (including Figs 1–3 and S2–S3) to improve clarity, consistency, and interpretability of presentation.

Several table titles, notes, and explanatory descriptions were also refined for clarity. No underlying analyses or results were changed.

Response to Comment 4A and B:
We refer the reviewer to our response to Reviewer #1, Comment 1 above, where we address the distinction between the five- and six-factor solutions and further clarify the role and interpretation of transformational leadership within the SFSS model.

**Reviewer #4**

*1) I think you should partly rewrite your motivation. Your critique for the literature that tries to predict the drivers of founding a startup is too harsh. You cannot blame them for not doing what they do not intend to do. You want to predict success conditional on entering into entrepreneurship. That is wonderful but that is another question, not the right question.*

*2) Similarly, one could criticize you for focusing on individual level driver while success is obviously dependent on collaboration and on environmental variables (quality of the team, availability of credit etc). So maybe you should strengthen the psychological part of your motivation*

*3) I believe you should cite "Botelho, Tristan L., Daniel C. Fehder, and Yael V. Hochberg. 2026. "Innovation-Driven Entrepreneurship." Journal of Economic Literature 64 (1): 89–140.”*

*4) There are a number of methodological details that should be confined in the appendix, while it would be interesting to present and discuss a little bit the items you are using to elicit the various dimensions. I think you should report the phrasing in the text. E.g. why not reporting the wording of the items in Table 4?*

*5) I think it is very interesting how you make preliminary efforts through biographies and other grey sources but then you do not explain how practically you were guided by this material*

Response to Comment 1:
We have now revised several conceptual framing passages in the Abstract (p. 2), Introduction (pp. 3-9), Discussion (p. 37), and Conclusion (p. 47) to more clearly distinguish entrepreneurial entry frameworks from founder-success research. In the revised manuscript, entrepreneurial personality and intention models are now described as valuable approaches for understanding entrepreneurial entry, while the present study is positioned more narrowly around founder-related characteristics associated with realized startup success. We also softened several formulations that could be interpreted as overly deterministic or comparative, including references to “high-potential founders” and statements implying that existing models “underperform”.

Section 1.1 (“Conceptual Contribution”, pp. 10-11) was revised to clarify that the present study focuses on explanatory specificity within the context of realized startup outcomes rather than proposing a competing general theory of entrepreneurship. We also revised several passages referring to “startup success” toward more cautious wording referring to characteristics “associated with realized startup success.”

Response to Comment 2:

We agree that startup success is influenced not only by founder-level characteristics, but also by broader organizational, collaborative, financial, and ecosystem-level conditions. In the revised manuscript, we strengthened the psychological framing of the study by more clearly positioning founder characteristics as one important level of analysis within a broader multi-level entrepreneurial framework, rather than as deterministic or sufficient causes of startup success.

Specifically, the Introduction and Discussion now more explicitly situate founder-related characteristics within processes of decision-making under uncertainty, resource orchestration, team mobilization, and venture execution, while the Limitations section clarifies that startup success also depends on organizational, financial, and ecosystem conditions beyond the individual founder (p. 45).

Response to Comment 3:

We included the suggested reference (Botelho et al., 2026) in the Introduction (p. 4) to better contextualize the distinction between innovation-driven entrepreneurship and broader forms of entrepreneurial activity. The reference was also added to the References list.

Response to Comment 4:

We refer the reviewer to our response to Reviewer #1, Comment 3 above, where we note that representative example items are now included to help better illustrate the dimensions described in Table 4.

We also reviewed the manuscript for opportunities to reduce excessive methodological detail in the main text and improve overall readability.

Response to Comment 5:

We refer the reviewer to our response to Reviewer #1, Comment 3 above, where we describe revisions to Section 2.2 clarifying how founder autobiographies, practitioner interviews, investor perspectives, and related founder materials informed dimensional development and initial item generation.

**Reviewer #5:**

*After a careful review, I acknowledge that the statistical analysis is coherent and appropriately performed. Furthermore, line 611 (Section 3.2, Interpreting the Extracted Factors) is quite eloquently presented, which makes complex concepts intelligible.*
