# Supplementary material for: Development and validation of a domain-specific scale of founder characteristics associated with startup success
Source: PLoS One. 2026 Jun 26;21(6):e0351970. doi: 10.1371/journal.pone.0351970 (PMC13308860; doi:10.1371/journal.pone.0351970)
Supplement: Peer review history

# Peer review: Decision Letter 0

**DOI:** 10.1371/journal.pone.0351970.r001

**Authors:** Marco Arraya

14 Apr 2026

PONE-D-26-04583

Development and validation of a domain-specific scale of founder characteristics associated with startup success

PLOS One

Dear Dr. C. Danilovska,

Thank you for submitting your manuscript to PLOS ONE. After careful consideration, we feel that it has merit but does not fully meet PLOS ONE’s publication criteria as it currently stands. Therefore, we invite you to submit a revised version of the manuscript that addresses the points raised during the review process.

Please submit your revised manuscript by May 29 2026 11:59PM. If you will need more time than this to complete your revisions, please reply to this message or contact the journal office at plosone@plos.org. When you're ready to submit your revision, log on to https://www.editorialmanager.com/pone/ and select the 'Submissions Needing Revision' folder to locate your manuscript file.

Please include the following items when submitting your revised manuscript:

- A letter that responds to each point raised by the academic editor and reviewer(s). You should upload this letter as a separate file labeled 'Response to Reviewers'.
- A marked-up copy of your manuscript that highlights changes made to the original version. You should upload this as a separate file labeled 'Revised Manuscript with Track Changes'.
- An unmarked version of your revised paper without tracked changes. You should upload this as a separate file labeled 'Manuscript'.

If you would like to make changes to your financial disclosure, please include your updated statement in your cover letter. Guidelines for resubmitting your figure files are available below the reviewer comments at the end of this letter.

If applicable, we recommend that you deposit your laboratory protocols in protocols.io to enhance the reproducibility of your results. Protocols.io assigns your protocol its own identifier (DOI) so that it can be cited independently in the future. For instructions see: https://journals.plos.org/plosone/s/submission-guidelines#loc-laboratory-protocols. Additionally, PLOS ONE offers an option for publishing peer-reviewed Lab Protocol articles, which describe protocols hosted on protocols.io. Read more information on sharing protocols at https://plos.org/protocols?utm_medium=editorial-email&utm_source=authorletters&utm_campaign=protocols.

As the corresponding author, your ORCID iD is verified in the submission system and will appear in the published article. PLOS supports the use of ORCID, and we encourage all coauthors to register for an ORCID iD and use it as well. Please encourage your coauthors to verify their ORCID iD within the submission system before final acceptance, as unverified ORCID iDs will not appear in the published article. *Only*  the individual author can complete the verification step; PLOS staff *cannot*  verify ORCID iDs on behalf of authors.

We look forward to receiving your revised manuscript.

Kind regards,

Marco Arraya, Ph.D

Academic Editor

PLOS One

**Journal Requirements:**

When submitting your revision, we need you to address these additional requirements.

1. Please ensure that your manuscript meets PLOS ONE's style requirements, including those for file naming. The PLOS ONE style templates can be found at

https://journals.plos.org/plosone/s/file?id=wjVg/PLOSOne_formatting_sample_main_body.pdf and

https://journals.plos.org/plosone/s/file?id=ba62/PLOSOne_formatting_sample_title_authors_affiliations.pdf

2. In the online submission form you indicate that your data is not available for proprietary reasons and have provided a contact point for accessing this data. Please note that your current contact point is a co-author on this manuscript. According to our Data Policy, the contact point must not be an author on the manuscript and must be an institutional contact, ideally not an individual. Please revise your data statement to a non-author institutional point of contact, such as a data access or ethics committee, and send this to us via return email. Please also include contact information for the third party organization, and please include the full citation of where the data can be found.

3. Your ethics statement should only appear in the Methods section of your manuscript. If your ethics statement is written in any section besides the Methods, please delete it from any other section.

4. Please upload a new copy of Figures 1- 3 and Supporting Figures 1-3 as the detail is not clear. Please follow the link for more information:  https://journals.plos.org/plosone/s/figures

5. We notice that your supplementary figures are uploaded with the file type 'Figure'. Please amend the file type to 'Supporting Information'. Please ensure that each Supporting Information file has a legend listed in the manuscript after the references list.

6. If the reviewer comments include a recommendation to cite specific previously published works, please review and evaluate these publications to determine whether they are relevant and should be cited. There is no requirement to cite these works unless the editor has indicated otherwise.

7. Please review your reference list to ensure that it is complete and correct. If you have cited papers that have been retracted, please include the rationale for doing so in the manuscript text, or remove these references and replace them with relevant current references. Any changes to the reference list should be mentioned in the rebuttal letter that accompanies your revised manuscript. If you need to cite a retracted article, indicate the article’s retracted status in the References list and also include a citation and full reference for the retraction notice.

**Additional Editor Comments:**

I congratulate you on the work you have done so far, however, your research needs more work. I suggest you read the reviewers' contributions and act accordingly. In addition to the fact that in my opinion the study will improve with more work in the Implications section.

[Note: HTML markup is below. Please do not edit.]

Reviewers' comments:

Reviewer's Responses to Questions

**Comments to the Author**

1. Is the manuscript technically sound, and do the data support the conclusions?

The manuscript must describe a technically sound piece of scientific research with data that supports the conclusions. Experiments must have been conducted rigorously, with appropriate controls, replication, and sample sizes. The conclusions must be drawn appropriately based on the data presented.

Reviewer #1: Yes

Reviewer #2: Yes

Reviewer #3: Partly

Reviewer #4: Yes

Reviewer #5: Yes

**********

2. Has the statistical analysis been performed appropriately and rigorously?

Reviewer #1: Yes

Reviewer #2: Yes

Reviewer #3: Yes

Reviewer #4: Yes

Reviewer #5: Yes

**********

3. Have the authors made all data underlying the findings in their manuscript fully available?

The PLOS Data policy requires authors to make all data underlying the findings described in their manuscript fully available without restriction, with rare exception (please refer to the Data Availability Statement in the manuscript PDF file). The data should be provided as part of the manuscript or its supporting information, or deposited to a public repository. For example, in addition to summary statistics, the data points behind means, medians and variance measures should be available. If there are restrictions on publicly sharing data—e.g. participant privacy or use of data from a third party—those must be specified.

Reviewer #1: Yes

Reviewer #2: No

Reviewer #3: Yes

Reviewer #4: Yes

Reviewer #5: Yes

**********

4. Is the manuscript presented in an intelligible fashion and written in standard English?

PLOS ONE does not copyedit accepted manuscripts, so the language in submitted articles must be clear, correct, and unambiguous. Any typographical or grammatical errors should be corrected at revision, so please note any specific errors here.

Reviewer #1: Yes

Reviewer #2: Yes

Reviewer #3: Yes

Reviewer #4: Yes

Reviewer #5: Yes

**********

5. Review Comments to the Author

Please use the space provided to explain your answers to the questions above. You may also include additional comments for the author, including concerns about dual publication, research ethics, or publication ethics. (Please upload your review as an attachment if it exceeds 20,000 characters)

Reviewer #1: Overall, this is a promising and potentially high-impact scale development paper. I recommend focusing revisions on (i) clarifying the factor solution and aligning claims, (ii) addressing measurement invariance (or appropriately tempering group comparison interpretations), and (iii) improving transparency for reproducibility and practical use.

Reviewer #2: This manuscript is well structure. It is methodologically sound and focus on domain specific scale that measure founder characteristics that are further linked to startup success. The topic is mainly relevant to entrepreneurship research and contribute to organizational research. The study meaningful academic value. The sample size is very large so the systematic scale development was done. Expert review was use. Exploratory and confirmatory factor analysis, reliability testing is used. The methods strengthen the credibility of study. However, some claims about predictive utility are strong but these claims are to be interpreted cautiously. For future longitudinal data can be used for validation. Data availability is data can be shared through the public repositories; the anonymised datasets are recommended for sharing. Over all the study is strong and suitable for publication after minor revisions.

Reviewer #3: Minor Comments:

1. Abstract: Well written

2. Manuscript is generally well written but:

A. Some sections are overly long (especially introduction)

B. Repetition in discussion

Scope for reducing the word count

3. Tables and figures should be simplified.

4. Clarify:

A. Difference between 5-factor vs 6-factor model

B. Role of leadership more explicitly

5. Reference: Ok

Reviewer #4: 1) I think you should partly rewrite your motivation. Your critique for the literature that tries to predict the drivers of founding a startup is too harsch. You cannot blame them for not doing what they do not intend to do. You want to predict success conditional on entering into entrepreneurship. That is wonderful but that is another question, not the right question.

2) Similarly, one could criticize you for focusing on individual level driver while success is obviously dependent on collaboration and on environmental variables (quality of the team, availability of credit etc). So maybe you should strengthen the psychological part of your motivation

3) I believe you should cite "Botelho, Tristan L., Daniel C. Fehder, and Yael V. Hochberg. 2026. "Innovation-Driven Entrepreneurship." Journal of Economic Literature 64 (1): 89–140."

4) There are a number of methodological details that should be confined in the appendix, while it would be interesting to present and discuss a little bit the items you are using to elicit the various dimensions. I think you should report the phrasing in the text. E.g. why not reporting the wording of the items in Table 4?

5) I think it is very interesting how you make preliminary efforts through biographies and other grey sources but then you do not explain how practically you were guided by this material

Reviewer #5: After a careful review, I acknowledge that the statistical analysis is coherent and appropriately performed. Furthermore, line 611 (Section 3.2, Interpreting the Extracted Factors) is quite eloquently presented, which makes complex concepts intelligible.

**********

6. PLOS authors have the option to publish the peer review history of their article (what does this mean?). If published, this will include your full peer review and any attached files.

If you choose “no”, your identity will remain anonymous but your review may still be made public.

**Do you want your identity to be public for this peer review?**  For information about this choice, including consent withdrawal, please see our Privacy Policy.

Reviewer #1: **Yes:**  Nath Amornpinyo

Reviewer #2: **Yes:**  Sunbul Naeem Cheema

Reviewer #3: **Yes:**  Rajib Bora

Reviewer #4: No

Reviewer #5: No

**********

[NOTE: If reviewer comments were submitted as an attachment file, they will be attached to this email and accessible via the submission site. Please log into your account, locate the manuscript record, and check for the action link "View Attachments". If this link does not appear, there are no attachment files.]

To ensure your figures meet our technical requirements, please review our figure guidelines: https://journals.plos.org/plosone/s/figures

You may also use PLOS’s free figure tool, NAAS, to help you prepare publication quality figures: https://journals.plos.org/plosone/s/figures#loc-tools-for-figure-preparation.

NAAS will assess whether your figures meet our technical requirements by comparing each figure against our figure specifications.

Attachment Submitted filename: Minor Comments.docx

# Author comment: Author response to Decision Letter 1

**DOI:** 10.1371/journal.pone.0351970.r002

29 May 2026

We thank the Reviewers and the Managing Editor for their careful and constructive evaluations of the manuscript. Below, we address both the reviewer comments and the additional editorial recommendations, and believe the revisions have improved the manuscript’s clarity, transparency, and interpretability while preserving the core structure and contribution of the SFSS framework.

In response to the reviews and editorial comments, we have: (i) clarified the conceptual distinction between the five- and six-factor solutions and aligned related claims throughout the manuscript; (ii) conducted additional measurement invariance analyses across Successful Startup Founders, Corporate Managers, and Aspiring Entrepreneurs; (iii) refined wording throughout the manuscript to better distinguish discriminative validity from predictive validity and to avoid overstating implications of the cross-sectional design; (iv) clarified and expanded selected methodological, interpretative, and practical implication sections; and (v) updated the Data Availability statement to clarify procedures for accessing de-identified data.

We also describe below how we addressed the Journal Requirements and additional editorial recommendations. Journal Requirements, reviewer comments, and editorial comments are reproduced below in italics, followed by our responses in regular text. All manuscript changes are shown using track changes.

Response to Journal Requirements

1. PLOS ONE formatting requirements

Response to Point 1: The title page, manuscript, figures, supporting information files, file naming conventions, and section titles were reviewed and updated to comply with PLOS ONE formatting requirements and submission guidelines.

2. Data availability statement and institutional contact point

Response to Point 2: Response to Point 2: The Data Availability Statement was revised to replace the previous author-based contact with a non-author institutional contact point, in accordance with PLOS ONE Data Policy requirements. The revised statement now includes the OSF repository citation, the institutional contact information for data access requests, and details regarding access to the de-identified dataset under institutional oversight.

3. Ethics statement placement

Response to Point 3: The ethics statement was reviewed and retained only within the Methods section of the manuscript, in accordance with journal requirements.

4. Figure quality

Response to Point 4: New high-resolution versions of Figures 1–3 and Supporting Figures 1–3 were prepared and uploaded in accordance with PLOS ONE figure requirements.

5. Supporting Information file types and legends

Response to Point 5: Supporting figures were re-uploaded using the correct “Supporting Information” file designation. Corresponding legends for all Supporting Information files were added to the manuscript after the reference list.

6. Reviewer-suggested references

Response to Point 6: The reviewer-suggested publication was reviewed, found relevant to the present study, and incorporated into the revised manuscript.

7. Reference list review

Response to Point 7: The reference list was reviewed for completeness, accuracy, and compliance with PLOS ONE formatting requirements. No retracted references are included in the revised manuscript. In addition, in-text citations and the reference list were revised to conform to PLOS ONE’s Vancouver citation style, including conversion of author–date citations to numbered citations where applicable.

Additional Editor Comments:I congratulate you on the work you have done so far, however, your research needs more work. I suggest you read the reviewers' contributions and act accordingly. In addition to the fact that in my opinion the study will improve with more work in the Implications section.

Response to Editor Comment:

We revised Section 4.8 (“Practical implications”, pp.44-45) to strengthen the connection between the study findings and their potential implications for entrepreneurial finance, founder development, and innovation policy. The revised section now more clearly distinguishes founder characteristics associated with realized startup outcomes from entrepreneurial intent alone, clarifies potential applications of the SFSS framework in evaluation, mentoring, training, and ecosystem-support contexts, and includes more concrete examples linked to specific SFSS dimensions (e.g., opportunity-recognition training or strategic-planning support). We also refined the wording throughout the section to better define the intended scope and boundaries of interpretation and application.

Response to Reviewers Comments

Reviewer #1

Overall, this is a promising and potentially high-impact scale development paper. I recommend focusing revisions on:

(i) clarifying the factor solution and aligning claims.

(ii) addressing measurement invariance (or appropriately tempering group comparison interpretations), and

(iii) improving transparency for reproducibility and practical use.

Response to Comment 1:

We revised Section 4.3 (“Five or six factors?”, pp. 40–41) to clarify the conceptual and statistical distinction between the five- and six-factor solutions. The revised text now describes the five-factor structure as the more parsimonious cross-group representation of founder success characteristics, while positioning the six-factor structure as a theoretically motivated founder-specific extension that captures transformational leadership within entrepreneurial contexts. Within the same section, we also expanded the interpretation of transformational leadership to clarify its potentially context-dependent and developmentally expressed nature across venture stages, organizational complexity, and entrepreneurial contexts.

In addition, several passages throughout the manuscript were revised to better align interpretative claims with the underlying cross-sectional design. In particular, wording related to “founder selection” and “early identification of startup founders with high success potential” was revised toward more cautious phrasing focused on the structured evaluation and differentiation of founder-related characteristics.

Response to Comment 2:We conducted additional multi-group measurement invariance analyses across Successful Startup Founders (SSF), Corporate Managers (CM), and Aspiring Entrepreneurs (AE) using the final six-factor, 31-item model. Configural, metric, scalar, and strict invariance models were evaluated. The results supported substantial cross-group stability of the SFSS factor structure, with only modest changes in practical fit indices across increasingly constrained models. Although some deterioration in fit was observed at the scalar and strict levels, the overall pattern supported general comparability of the latent structure while also indicating some group-specific variation in item intercepts and residual variances.

A new subsection titled “3.1.3.1 Measurement Invariance Across Entrepreneurial and Comparison Groups” and a corresponding table reporting invariance fit indices were added to the Results section (pp. 28–29). We also added a brief methodological description of the invariance procedure in Section 2.5.3 (“Confirmatory factor analysis”, p. 22). Given the large sample size (N = 10,007), practical fit indices were prioritised when evaluating invariance models due to the known sensitivity of chi-square statistics under large-N conditions. Corresponding methodological references on measurement invariance were also added to the References list.

In addition, earlier statements indicating that measurement invariance analyses were deferred to future validation studies were removed or revised in Sections 2.1 and 2.2 to reflect the inclusion of the present invariance analyses.

Response to Comment 3:We revised several sections of the manuscript to improve clarity, interpretability, and practical usability of the SFSS framework and development process. Section 2.2 (“Dimensions identification and initial item generation”) was expanded to more clearly describe how founder autobiographies, practitioner interviews, investor perspectives, and related founder materials informed dimensional development and item generation (p. 16). Section 3.2 (“Interpreting the extracted factors”) was also revised to include representative example item wording for each factor to improve interpretability of the extracted dimensions (pp. 30–31).

In addition, as noted above in our response to Comment 1, we further clarified the distinction between the five- and six-factor solutions in Section 4.3 and revised several passages throughout the manuscript to more clearly distinguish discriminative validity from predictive validity. We also made several editorial and reporting updates throughout the manuscript, including revisions to the Data Availability statement in accordance with journal requirements (see also our response to the Journal Requirements).

Reviewer #2:

This manuscript is well structured. It is methodologically sound and focus on domain specific scale that measure founder characteristics that are further linked to startup success. The topic is mainly relevant to entrepreneurship research and contribute to organizational research. The study has meaningful academic value. The sample size is very large so the systematic scale development was done. Expert review was use. Exploratory and confirmatory factor analysis, reliability testing is used. The methods strengthen the credibility of study. Overall the study is strong and suitable for publication after minor revisions.

1. However, some claims about predictive utility are strong but these claims are to be interpreted cautiously.

2. For future longitudinal data can be used for validation.

3. Data availability is data can be shared through the public repositories; the anonymised datasets are recommended for sharing.

Response to Comment 1:

Several passages throughout the manuscript were revised to more clearly distinguish discriminative validity from predictive validity and to avoid overstating implications of the present cross-sectional design. Specifically, in the Abstract (p. 2) we changed “Early identification of startup founders with high success potential” to “Early differentiation of startup founders with high success potential” and revised references to “founder selection” toward more cautious wording focused on the structured evaluation of founder-related characteristics.

In the Introduction (pp. 3 and 5), several predictive and selection-oriented statements were softened, including replacing deterministic phrasing such as “can materially improve” with more cautious wording (“may improve”), revising references to “identifying high-potential startup founders,” and replacing the statement that limitations “can leave high-potential founders under-identified and resources misallocated” with wording indicating that such limitations “may contribute to founder profiles associated with strong venture outcomes being overlooked and resources being allocated inefficiently.”

Several conceptual statements in the Discussion were revised to avoid deterministic interpretations of founder characteristics and entrepreneurial outcomes (e.g., replacing “founder characteristics shape” with “founder characteristics are likely to influence”, p. 37).

In addition, the practical framing in the Conclusion was also revised by replacing wording referring to an “objective, early-stage assessment framework” supporting “founder selection” with more cautious language describing the SFSS as a “domain-specific framework for assessing founder-related characteristics” that may help inform founder evaluation and capital-allocation processes (pp. 47-48).

Response to Comment 2:

We agree that future longitudinal analyses will be important for further validation of the SFSS framework. The need for longitudinal validation was already acknowledged in the original manuscript, including in the Abstract, Introduction, Methodological Considerations, and Limitations/Future Directions sections. In the revised manuscript, several passages were further refined to more clearly distinguish discriminative validity from predictive validity and to avoid interpretations extending beyond the present cross-sectional design (see also our response to Comment 1 above).

For example, the revised Introduction now states that the SFSS provides “a foundation for future longitudinal studies” while emphasizing that the present work focuses on founder profiles associated with realized startup success rather than prospective prediction (p. 11).

Response to Comment 3:

We refer the reviewer to our response to the Journal Requirements (Point 2), where we describe revisions to the Data Availability statement addressing this issue.

Reviewer #3

Reviewer #3: Minor Comments:1. Abstract: Well written2. Manuscript is generally well written but:A. Some sections are overly long (especially introduction)B. Repetition in discussionScope for reducing the word count3. Tables and figures should be simplified.4. Clarify:A. Difference between 5-factor vs 6-factor modelB. Role of leadership more explicitly5. Reference: Ok

Response to Comment 2A and 2B:We have carefully revised the Introduction and Discussion sections to further improve conciseness, reduce repetition, and streamline several extended conceptual and methodological passages without changing any core conceptual arguments or interpretation of the findings.

Specifically, in the Introduction (pp. 3-11), we have shortened and consolidated discussions of prior entrepreneurial personality and orientation instruments, reduced repetitive comparisons between entrepreneurial intention and realized startup success, simplified operational definition sections, and condensed examples of heterogeneous entrepreneurial samples used in prior research.

In addition, in the Discussion (pp. 36-47), we have reduced repetition across sections discussing broad personality models, entrepreneurial intention frameworks, and founder-specific characteristics. We have also streamlined several interpretive passages, reduced repetitive effect-size interpretations, and shortened repeated practical implications related to scaling, founder evaluation, and resource allocation.

These revisions now help to reduce overall manuscript length while preserving the conceptual and theoretical contributions of the study.

Response to Comment 3:We reviewed all tables and figures for readability, presentation clarity, and potential simplification. Overall, the tables and figures were already relatively streamlined, and no major structural simplifications or analytical changes were introduced, as further reduction could risk loss of relevant methodological or interpretative information.

Nevertheless, several presentation refinements were implemented to improve clarity and readability. Figure 1 (scree plot) was revised to improve interpretability of the factor-retention criteria and corresponding visual reference markers. In addition, axis labels, legends, captions, and formatting details were added or refined across figures (including Figs 1–3 and S2–S3) to improve clarity, consistency, and interpretability of presentation.

Several table titles, notes, and explanatory descriptions were also refined for clarity. No underlying analyses or results were changed.

Response to Comment 4A and B:We refer the reviewer to our response to Reviewer #1, Comment 1 above, where we address the distinction between the five- and six-factor solutions and further clarify the role and interpretation of transformational leadership within the SFSS model.

Reviewer #4

1) I think you should partly rewrite your motivation. Your critique for the literature that tries to predict the drivers of founding a startup is too harsh. You cannot blame them for not doing what they do not intend to do. You want to predict success conditional on entering into entrepreneurship. That is wonderful but that is another question, not the right question.

2) Similarly, one could criticize you for focusing on individual level driver while success is obviously dependent on collaboration and on environmental variables (quality of the team, availability of credit etc). So may

Attachment Submitted filename: SFSS_PlosOne_LetterToReviewers_28May2026_ff.docx

# Editor report: Decision Letter 1

**DOI:** 10.1371/journal.pone.0351970.r003

**Authors:** Marco Arraya, Marco Arraya

3 Jun 2026

Development and validation of a domain-specific scale of founder characteristics associated with startup success

PONE-D-26-04583R1

Dear Dr. Irena,

We’re pleased to inform you that your manuscript has been judged scientifically suitable for publication and will be formally accepted for publication once it meets all outstanding technical requirements.

Within one week, you’ll receive an e-mail detailing the required amendments. When these have been addressed, you’ll receive a formal acceptance letter and your manuscript will be scheduled for publication.

An invoice will be generated when your article is formally accepted. Please note, if your institution has a publishing partnership with PLOS and your article meets the relevant criteria, all or part of your publication costs will be covered. Please make sure your user information is up-to-date by logging into Editorial Manager at Editorial Manager® and clicking the ‘Update My Information' link at the top of the page. For questions related to billing, please contact billing support.

If your institution or institutions have a press office, please notify them about your upcoming paper to help maximize its impact. If they’ll be preparing press materials, please inform our press team as soon as possible -- no later than 48 hours after receiving the formal acceptance. Your manuscript will remain under strict press embargo until 2 pm Eastern Time on the date of publication. For more information, please contact onepress@plos.org.

Kind regards,

Marco  Arraya, Ph.D

Academic Editor

PLOS One

Additional Editor Comments (optional):

Reviewers' comments:

# Editor report: Acceptance letter

**DOI:** 10.1371/journal.pone.0351970.r004

**Authors:** Marco Arraya, Marco Arraya

PONE-D-26-04583R1

PLOS One

Dear Dr. C. Danilovska,

I'm pleased to inform you that your manuscript has been deemed suitable for publication in PLOS One. Congratulations! Your manuscript is now being handed over to our production team.

At this stage, our production department will prepare your paper for publication. This includes ensuring the following:

* All references, tables, and figures are properly cited

* All relevant supporting information is included in the manuscript submission,

* There are no issues that prevent the paper from being properly typeset

You will receive further instructions from the production team, including instructions on how to review your proof when it is ready. Please keep in mind that we are working through a large volume of accepted articles, so please give us a few days to review your paper and let you know the next and final steps.

Lastly, if your institution or institutions have a press office, please let them know about your upcoming paper now to help maximize its impact. If they'll be preparing press materials, please inform our press team within the next 48 hours. Your manuscript will remain under strict press embargo until 2 pm Eastern Time on the date of publication. For more information, please contact onepress@plos.org.

You will receive an invoice from PLOS for your publication fee after your manuscript has reached the completed accept phase. If you receive an email requesting payment before acceptance or for any other service, this may be a phishing scheme. Learn how to identify phishing emails and protect your accounts at https://explore.plos.org/phishing.

If we can help with anything else, please email us at customercare@plos.org.

Thank you for submitting your work to PLOS ONE and supporting open access.

Kind regards,

PLOS ONE Editorial Office Staff

on behalf of

Dr. Marco António Mexia Arraya

Academic Editor

PLOS One
